# Structure Elucidation and Characterization of Novel Glycolipid Biosurfactant Produced by *Rouxiella badensis* DSM 100043^T^

**DOI:** 10.3390/molecules30081798

**Published:** 2025-04-17

**Authors:** Andre Fahriz Perdana Harahap, Jürgen Conrad, Mario Wolf, Jens Pfannstiel, Iris Klaiber, Jakob Grether, Eric Hiller, Maliheh Vahidinasab, Hanna Salminen, Chantal Treinen, Elvio Henrique Benatto Perino, Rudolf Hausmann

**Affiliations:** 1 Department of Bioprocess Engineering (150k), Institute of Food Science and Biotechnology, University of Hohenheim, Fruwirthstr. 12, 70599 Stuttgart, Germany; andrefahrizperdana.harahap@uni-hohenheim.de (A.F.P.H.); jakob.grether@uni-hohenheim.de (J.G.); eric.hiller@uni-hohenheim.de (E.H.); malihe.vahidinasab@uni-hohenheim.de (M.V.); eperino@uni-hohenheim.de (E.H.B.P.); 2 Department of Organic Chemistry (130b), Institute of Chemistry, University of Hohenheim, Garbenstr. 30, 70599 Stuttgart, Germany; juergen.conrad@uni-hohenheim.de (J.C.); wolf.mario@uni-hohenheim.de (M.W.); 3 Mass Spectrometry Unit, Core Facility Hohenheim, University of Hohenheim, Ottilie-Zeller-Weg 2, 70599 Stuttgart, Germany; jens.pfannstiel@uni-hohenheim.de (J.P.); iris.klaiber@uni-hohenheim.de (I.K.); 4 Department of Food Material Science (150g), Institute of Food Science and Biotechnology, University of Hohenheim, Garbenstr. 21/25, 70599 Stuttgart, Germany; hanna.salminen@uni-hohenheim.de; 5 Cellular Agriculture, TUM School of Life Sciences, Technical University of Munich, 85354 Freising, Germany; chantal.treinen@tum.de

**Keywords:** *Rouxiella badensis*, biosurfactants, emulsification, glucose–lipid, glycolipid, structure elucidation

## Abstract

Microbial biosurfactants have become increasingly attractive as promising ingredients for environmentally friendly products. The reasons for this are their generally good performance and biodegradability, low toxicity, production from renewable raw materials, and benefits for the environment perceived by consumers. In this study, we investigated the chemical structure and properties of a novel glycolipid from a new biosurfactant-producing strain, *Rouxiella badensis* DSM 100043^T^. Bioreactor cultivation was performed at 30 °C and pH 7.0 for 28 h using 15 g/L glycerol as a carbon source. The glycolipid was successfully purified from the ethyl acetate extract of the supernatant using medium pressure liquid chromatography (MPLC). The structure of the glycolipid was determined by one- and two-dimensional (^1^H and ^13^C) nuclear magnetic resonance (NMR) and confirmed by liquid chromatography electrospray ionization mass spectrometry (LC-ESI/MS). NMR analysis revealed the hydrophilic moiety as a glucose molecule and the hydrophobic moieties as 3-hydroxy-5-dodecenoic acid and 3-hydroxydecanoic acid, which are linked with the glucose by ester bonds at the C2 and C3 positions. Surface tension measurement with tensiometry indicated that the glucose–lipid could reduce the surface tension of water from 72.05 mN/m to 24.59 mN/m at 25 °C with a very low critical micelle concentration (CMC) of 5.69 mg/L. Moreover, the glucose–lipid demonstrated very good stability in maintaining emulsification activity at pH 2–8, a temperature of up to 100 °C, and a NaCl concentration of up to 15%. These results show that *R. badensis* DSM 100043^T^ produced a novel glycolipid biosurfactant with excellent surface-active properties, making it promising for further research or industrial applications.

## 1. Introduction

Microbial biosurfactants have attracted increasing interest in recent years [1]. Compared to traditional surfactants, microbial biosurfactants are often reported to offer several benefits, including low environmental toxicity, excellent biodegradability, and derivation from renewable raw materials. Some microbial biosurfactants also strongly display desired performance characteristics such as foaming, low CMC, efficient emulsification, and strong reduction in interfacial tension. These properties increase their effectiveness in a range of applications [2,3].

Glycolipids are among the most frequently used low-molecular-weight biosurfactants, and their potential applications have been extensively investigated. Glycolipids are a diverse class of lipids that contain a carbohydrate moiety covalently linked to a lipid component, which can be a fatty acid, an alkyl chain, or another hydrophobic group, typically via a glycosidic or ester bond. The variety of glycolipids is due to differences in the lipid chains and carbohydrate moiety; this subclass includes rhamnolipids, sophorolipids, mannosylerythritol lipids, cellobiose lipids, trehalolipids, xylolipids, and glucose–lipids [4].

Studies focusing on investigation of *R. badensis*’s potential are still very limited. *R. badensis* strain SER3 was isolated from the phyllosphere of strawberry fruit and showed antifungal activity against postharvest fungal pathogens of berries [5]. Another study reported that *R. badensis acadiensis Canan* (*R. acadiensis*), isolated from the blueberry biota, has an antibacterial effect, and possesses characteristics of a probiotic strain on the mammalian intestinal ecosystem [6].

*R. badensis*-type strain DSM 100043^T^ was first isolated from the upper layer of a raised bog of the northern Black Forest, Germany. ^1^H correlation spectroscopy (COSY), ^13^C heteronuclear single quantum coherence (HSQC), and ^13^C heteronuclear multiple bond correlation (HMBC) NMR spectroscopy revealed that this strain produced several forms of free fatty acid, such as 3′ hydroxyl lauroleic acid, myristic acid, and myristoleic acid. Hydrophilic moieties of glycolipid mixtures produced by this strain revealed the presence of glucose and two forms of talopyranose without further determination of ester or ether bonds existing at the acylation sites [7].

This study investigated the complete structure of glycolipid biosurfactants produced by *R. badensis* DSM 100043^T^ by utilizing two-dimensional NMR spectroscopy and LC-ESI/MS after performing extraction and purification from fermentation broth of bioreactor cultivation. In addition, characterization of the surfactant properties of the glycolipid produced by *R. badensis* DSM 100043^T^ are reported.

## 2. Results and Discussion

### 2.1. Shake Flask and Bioreactor Cultivation

To gain understanding of cell growth and glucose–lipid production by *R. badensis* DSM 100043^T^, shake flask cultivations were first conducted in defined mineral salt medium (MSM) using different temperatures and glycerol concentrations for 32 h. Previous studies on the cultivation of *R. badensis* DSM 100043^T^ were used as references for its growth condition in shake flask cultivation [7,8]. Conversion factors between cell dry weight (CDW) (g/L) and optical density were determined as 2.81 and 2.82 for shake flask and bioreactor cultivation, respectively (Tables S1 and S2). Using quantitative HPTLC measurement, the glucose–lipid could be detected and measured, allowing the time course of glucose–lipid production to be developed. The method for glucose–lipid quantification by HPTLC had been validated according to US FDA Guidance (1999), and the results can be seen in Supplementary Materials SM2 [9]. All shake flask cultivation graphs of *R. badensis* DSM 100043^T^ at different temperatures and glycerol concentrations can be found in Figure S1.

**Figure 1:**
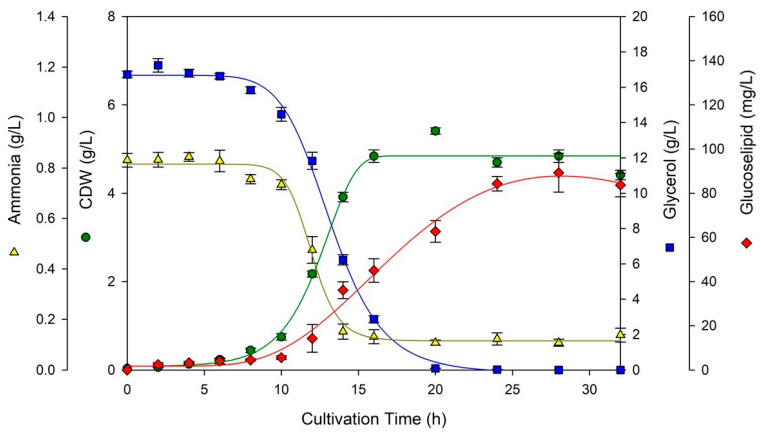
Time course of shake flask cultivation of *R. badensis* DSM 100043^T^ at 30 °C and 15 g/L glycerol for 32 h. Glycerol (blue square), glucose–lipid (red diamond), ammonia (yellow triangle), and CDW (green circle) were measured as biological triplicate.

Based on the temperature effect, an increase in temperature from 20 °C to 30 °C led to faster glycerol consumption. *R. badensis* DSM 100043^T^ showed growth limitation before glycerol was completely consumed, indicating possible additional substrate limitation aside from glycerol [10]. Interestingly, cultivation at 30 °C resulted in the lowest CDW but reached the highest glucose–lipid titer of 89.3 mg/L, which was higher than the titer achieved at 20 °C and 25 °C, as shown in Figure 1 and Figure S1. Nevertheless, a slight decrease in glucose–lipid titer was observed after 32 h of cultivation at 30 °C. The exact reason for this phenomenon remains unclear, but it may have been caused by the breakdown of glucose–lipid. Glucose–lipid breakdown may occur due to the activity of hydrolytic enzymes secreted by cells when the main carbon source is depleted; thus, the fed-batch cultivation mode would be an interesting subject of future investigations [11].

Based on the glycerol concentration effect, an increase in glycerol concentration from 10 g/L to 30 g/L led to slower glycerol consumption. When using 30 g/L glycerol, about 12 g/L glycerol remained at the end of cultivation, whereas the cells had already reached stationary phase by 12 h. Increasing glycerol concentration led to a decrease in growth rate, as can be seen in Table 1, which might have been caused by substrate inhibition. The highest glucose–lipid titer at 20 g/L glycerol was 55.9 mg/L after 28 h of cultivation. Increasing glycerol concentration to 30 g/L did not result in a higher titer of glucose–lipid. Overall, the cultivation of *R. badensis* DSM 100043^T^ at 30 °C and 15 g/L glycerol resulted in the highest glucose–lipid titer and productivity; thus, these growth conditions were applied in bioreactor cultivation.

**Figure 2:**
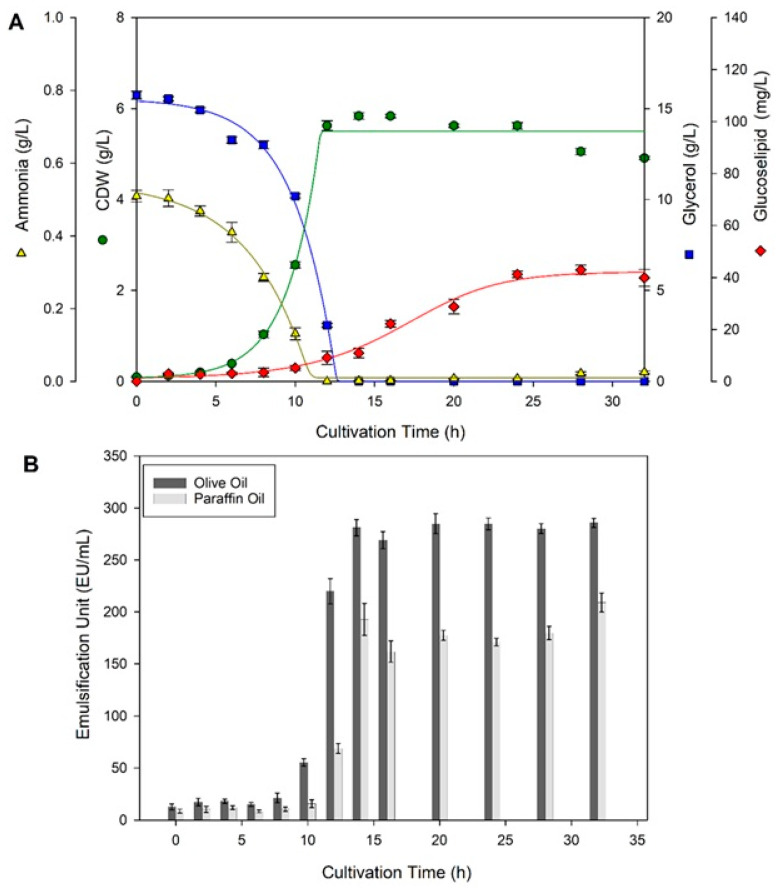
Time course of bioreactor cultivation of *R. badensis* DSM 100043^T^ at 30 °C and 15 g/L glycerol for 32 h, where glycerol (blue squares), glucose–lipid (red diamond), ammonia (yellow triangle), and CDW (green circle) were measured twice (**A**). Time course of emulsification units in olive oil (dark grey bar) and paraffin oil (light grey bar) of supernatants obtained from bioreactor cultivation (**B**).

The main objective of *R. badensis* DSM 100043^T^ bioreactor cultivation in this study was to obtain as much glycolipid as possible for further purification and structure elucidation; thus, the most optimum growth conditions in shake flask cultivation were implemented. As shown in Figure 2A, glycerol (15 g/L) was completely consumed after 14 h, while stationary phase was reached after 12 h cultivation. From Table 1, it seems that the bioreactor cultivation had relatively the same growth rate parameters as shake flask cultivation at 30 °C and 15 g/L glycerol. The lag phase in the current study was much shorter (less than 10 h) than in the bioreactor cultivation conducted in the previous study (more than 40 h) by Kügler et al. (2015) [7]. From Figure 1 and Figure 2A, it can be observed that the half values of glycerol consumption and growth curve are mostly the same, except for the delayed formation of glucose–lipid production, which might suggest the involvement of multiple enzymes for the synthesis of glucose–lipid. These enzymes likely became active or induced as the microbe switched from primary to secondary metabolism under nutrient limitation or stress conditions. With respect to the low glucose–lipid titer, future studies may focus on bioreactor optimization and upstream strategies to enhance production levels.

In this study, antifoam was added to the medium prior to inoculation to prevent living cells from being trapped in the foam. In addition, the dissolved oxygen level was maintained at a minimum of 20% to meet the required oxygen requirements of the growing cells. These two treatments might synergistically play a role in shortening the lag phase in bioreactor cultivation of *R. badensis* DSM 100043^T^. During the bioreactor cultivation, an excessive amount of foam started to build up at 12 h cultivation time, leading to the injection of 6 mL antifoam in total. This antifoam was tested and was found not to interfere with detection and purification of the glucose–lipid due to its insolubility in ethyl acetate during the extraction step. The formed foam indirectly indicated glucose–lipid production, as the emulsification units of the supernatant increased significantly at 12 h cultivation time, as shown in Figure 2B, due to its surfactant activity. After 14 h of cultivation, emulsification units of the supernatant remained constant for both paraffin oil and olive oil.

### 2.2. Purification and Structure Elucidation of Glucose–Lipid

For purification of glucose–lipid, 1.7 liters of fermentation broth from the bioreactor cultivation was centrifuged to obtain the yellowish-colored cell-free supernatant for subsequent extraction. The fermentation broth was harvested at 28 h of cultivation to avoid potential degradation of the glucose–lipid, which can be observed in the shake flask fermentation depicted in Figure 1. After extraction, evaporation of the organic solvents was performed using a rotary vacuum evaporator for 6 h to ensure complete removal of remaining ethyl acetate. This resulted in 118.2 mg of dark brownish crude extract with an oily texture. The crude extract was dissolved in 2 mL DMSO as an injection solvent for MPLC. The injection volume was kept as low as possible to minimize band spreading, peak broadening, and separation loss, thus improving separation between compounds in the crude extract, which led to higher purity of the targeted glucose–lipid. In this study, DMSO was by far the most effective solvent used for the injection of crude extract, as it could perfectly dissolve the crude extract. In addition, the high polarity of DMSO caused the glucose–lipid to interact with the C18 column immediately after sample injection rather than with DMSO.

**Figure 3:**
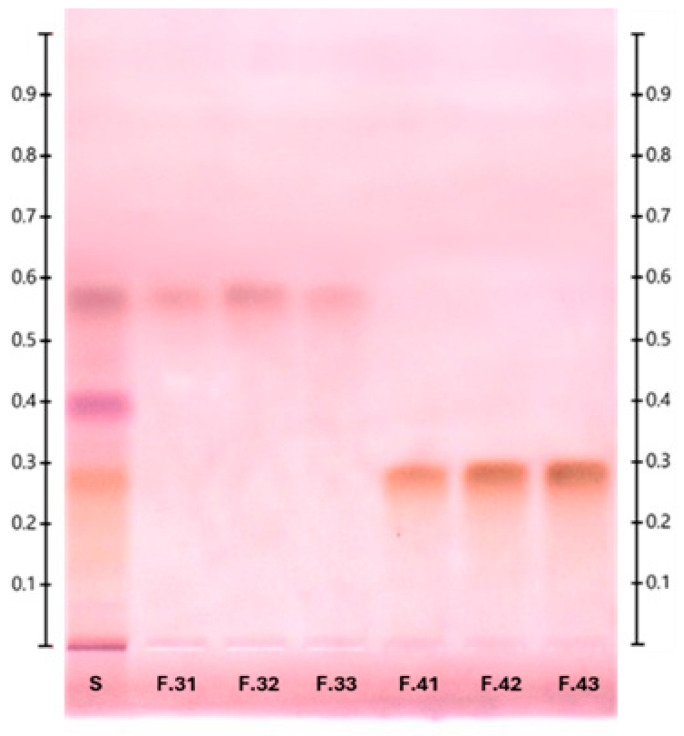
Vis-photograph of p-anisaldehyde-stained TLC silica gel 60 RP-18 F_254S_ plate showing MPLC-purified glucose–lipid successfully isolated from the crude extract sample (S) in fraction 41–43 at an *R_f_* value of 0.29. Fractions 31–33 at an *R_f_* value of 0.58 are non-targeted metabolites that showed no band during DPA derivatization, indicating that these fractions contain no carbohydrate moiety.

The target glucose–lipid eluted at 64% ACN in fractions 41–43, as shown on the TLC plate with an *R_f_* value of 0.29 in Figure 3. On the TLC plate, there were four different bands in the crude extract lane (S) with *R_f_* values of 0.01, 0.29, 0.40, and 0.58, indicating that there were at least four major components present in the ethyl acetate crude extract. Bands with *R_f_* values of 0.01 and 0.40 were washed away as flow-through together with the injection solvent at the very beginning of chromatography, indicating that there were no bindings with the C18 reverse-phase column. Fractions 31–33 were successfully isolated but showed no band on the TLC plate during derivatization with diphenylamine–aniline–phosphoric acid (DPA) reagent, indicating that these fractions did not contain any carbohydrate moiety [12]. The successfully purified glucose–lipid fractions 41–43 were finally pooled, and the solvent was evaporated to remove the remaining water–ACN mixture, which could have interfered with the NMR analysis. The total yield of dried glucose–lipid fractions 41–43 after purification was 37.4 mg, corresponding to 22 mg per liter of fermentation broth. The different value of the measured glucose–lipid concentration after 28 h of bioreactor fermentation (42.9 mg/L), as shown in Table 1, could have happened due to glucose–lipid loss during the extraction and purification steps.

**Figure 4:**
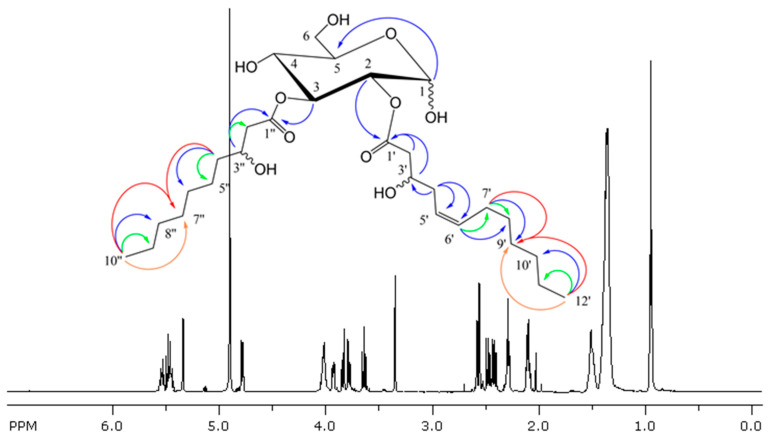
Chemical structure and ^1^H NMR spectrum of freshly dissolved glucose–lipid sample in methanol-d4. Arrows indicate important HMBC (blue), H2BC (green), HSQCTOCSY (orange), and band selective super-long-range HMBC (red) correlations.

The ^1^H NMR spectrum of freshly dissolved glucose–lipid sample showed typical proton signals of a carbohydrate of between *δ* 3.5 and 5.5 ppm and fatty acid moieties of between *δ* 0.9 and 2.6 ppm. Two additional proton signals at *δ* 4.01 and 4.02 ppm indicated two hydroxy substitutions, and two olefinic protons at *δ* 5.45 and 5.54 ppm indicated the presence of one double bond in the fatty acid residues (Figure 4).

**Figure 5:**
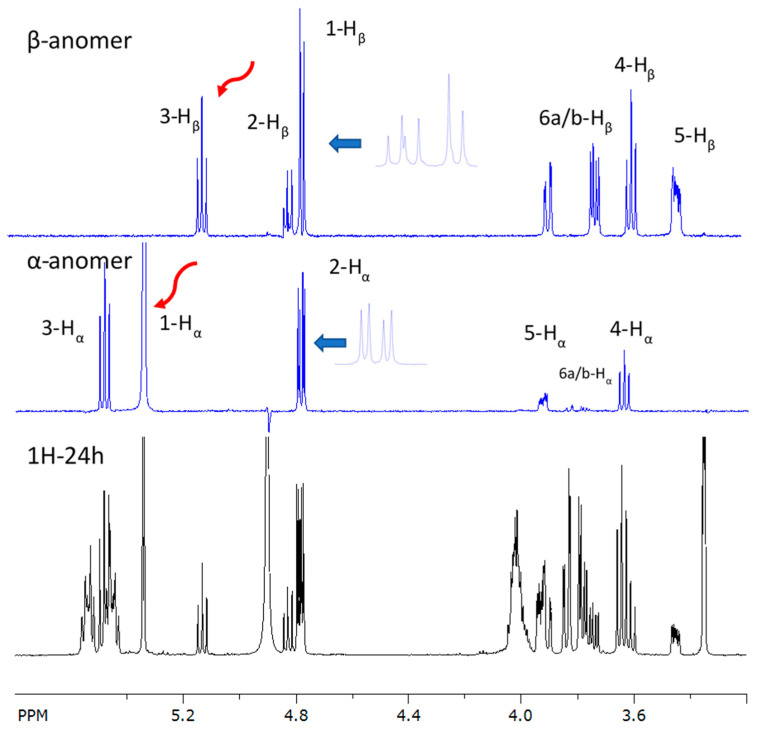
Expansion of ^1^H NMR (black) displaying sugar protons of glucose–lipid and 1D selective 1D TOCSYs (blue) of the α- and β-anomeric glucopyranosyl moieties. Sites of irradiation are marked by the red arrows. Blue arrows indicate expansions of the signals at δ 4.8 ppm.

In addition to these major signals, small proton signals of a minor component (ratio 94:6) that could not be chromatographically removed were observed. After about 24 h, an equilibrium mixture in the ratio of 73:27 (α-anomer (major): β-anomer (minor)) was established in methanol-*d*4, suggesting that the anomeric center was not acylated (Figures S2 and S3). Both anomeric sugar moieties consisted of five methines and one methylene each, as derived by detailed inspection of the COSY (Figure S4), F1-homodecoupled PSYCHE TOCSY (Figure S5), and selective 1D TOCSY (Figure 5). Analysis of the ^1^H coupling constants directly derived from the ^1^H NMR spectrum or in the case of signal overlap from selective 1D TOCSY (Figure 5) along with evaluation of the HSQC and HMBC spectra (Figures S6 and S7) revealed a glycopyranosyl moiety with a ^4^C_1_ conformation in the case of a D-configured glucose for the α- and β-anomeric forms, as shown in Figure 4 and Table 2 (NMR data).

The ^3^*J*_CH_ long-range correlations between downfield-shifted 2-H_α/β_ at *δ* 4.78/4.81ppm and carboxyl carbon C-1′_α/β_ at δ 172.65/172.30 ppm, as well as between downfield-shifted 3-H_α/β_ at δ 5.48/4.81ppm and carboxyl carbon C-1″_α/β_ at δ 173.06/172.99 ppm, indicated the presence of two fatty acid (FA) residues in the glucose–lipid. Further evaluation of standard COSY, HSQC, and HMBC established the same partial structure of the first four C atoms for both FAs in the major α-anomer, as shown in Figure 4. In the FA at C-3_α_, methylene 4″-H_α_ at δ 1.51 ppm showed a COSY cross-peak with diastereotopic methylene protons 5″-H_α_ at δ 1.47 and 1.39 ppm at C-5″_α_ and at δ 26.69 ppm, whereas in the FA at C-2_α_, methylene protons 4′-H_α_ at δ 2.28 ppm correlated with an olefinic 5′-H_α_ at δ 5.45 ppm and at C-5′_α_ at δ 125.95 ppm in the COSY spectrum. The latter shared a vicinal coupling of ^3^*J*_HH_ = 10.9 Hz with olefinic 6′-H_α_ at δ 5.54 ppm and at C-6′_α_ at *δ* 133.61 ppm, indicating a Z-configurated double bond. The adjacent aliphatic methylene 7′-H_α_ at *δ* 2.10 ppm and at C-7′_α_ at δ 28.42 ppm could be easily identified by COSY and HSQC. Both a ^3^*J*_CH_ long-range correlation between olefinic 6′-H_α_ and C-8′ in the band-selective HMBC (Figure S8) and a ^2^*J*_CH_ correlation between methylene 7′-H_α_ and C-8′ in the H2BC spectrum (Figure S9) established methylene C-8′ at δ 30.73 ppm. Moreover, a further ^3^*J*_CH_ correlation between 7′-H_α_ and methylene C-9′ at δ 30.12 ppm determined the 9′-position in the FA at C-2_α_ (Figure S8). The methylene C-6″ of the FA at C-3_α_ was unambiguously assigned at δ 30.71 ppm by the HMBC correlation between methylene 4″-H_α_ and C-6″. Due to strong signal overlap of the remaining aliphatic 5 methylene between δ 1.31 and 1.47 ppm and two terminal methyl groups at δ 0.93 ppm in the ^1^H NMR spectrum, more sophisticated NMR techniques were applied to unambiguously determine the chain length of each FA. To simplify signal assignment, a 1D PSYCHE spectrum was recorded to remove the multiplet structure of the ^1^H NMR signals and show only their chemical shifts as singlets [13,14,15,16]. Unfortunately, both terminal methyl groups exhibited the same ^13^C chemical shift at δ 14.45 ppm and ^1^H chemical shift at δ 0.93 ppm even in the PSYCHE spectrum (Figure S10). As such, the observed 2D correlations could only be treated in common and resulted in a terminal propyl group for each FA (C-10′–C-12′; C-8″–C10″). In the HSQCTOCSY spectrum with a mixing time of 100 ms (Figure S11), protons 7′-H_α_ showed a correlation with carbon C-10′ at δ 33 ppm of the terminal propyl moiety, indicating a total chain length of 12 carbons in the FA at C-2_α_. This finding is supported by a ^4^*J*_CH_ coupling of the terminal methyl group with C-9′ at δ 30.12 ppm in the band-selective super-long-range HMBC (Figure S12) [17,18]. Similarly, methylene carbon C-7″ at δ 30.48 ppm was assigned by HSQCTOCY and band-selective super-long-range HMBC (Figures S11 and S12) and revealed a carbon chain length of 10 for the FA at C-3_α_.

**Figure 6:**
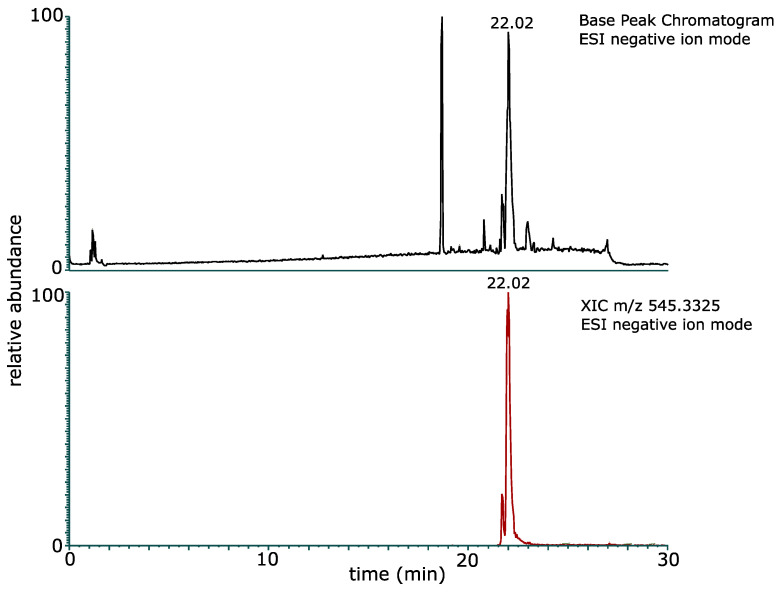
LC–ESI–MS/MS analysis of the purified glucose–lipid produced by *R. badensis* DSM 100043^T^: The base peak chromatogram in negative ion mode (**top panel**) and the extracted ion chromatogram (XIC) of *m*/*z* 545.3325 in negative ion mode (**bottom panel**) show an intense signal of the glucose–lipid at a retention time of 22.02 min.

**Figure 7:**
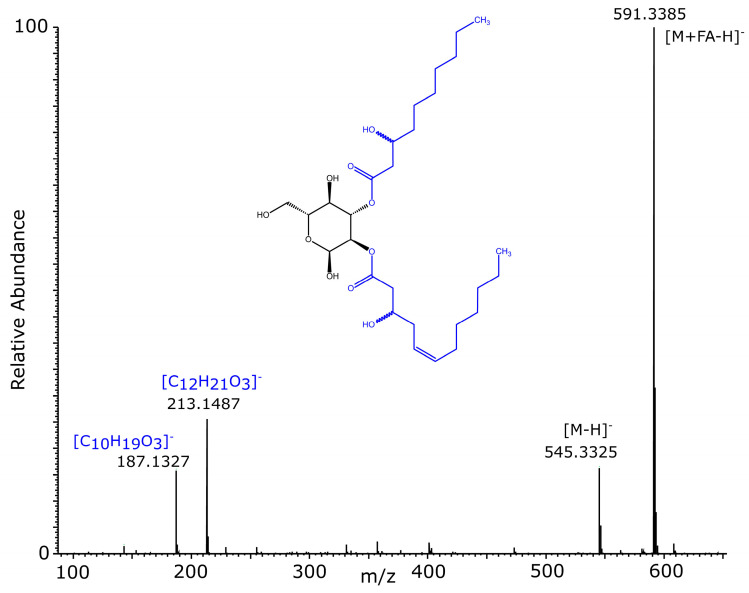
LC–ESI–MS/MS analysis of the purified glucose–lipid produced by *R. badensis* DSM 100043^T^: Mass spectrum in negative ion mode at a retention time of 22.02 min. The glucose–lipid was detected as a deprotonated molecular ion [M − H]^−^ and as a formic acid adduct [M + FA − H]^−^. In addition, in-source fragments of both fatty acids were detected. Sum formulas of the in-source fragment ions calculated from their exact *m*/*z* values agree well with the fatty acid structures of 3-hydroxy-5-dodecenoic acid (C12:1) and 3-hydroxydecanoic acid (C10:0). The inset shows the chemical structure of the glucose–lipid, with the fatty acids highlighted in blue.

In addition, the purified glucose–lipid sample was analyzed by high-resolution mass spectrometry. The base peak chromatogram of the LC–ESI–MS/MS analysis in negative ion mode showed an intense signal at 22.02 min (Figure 6). The mass spectrum obtained in negative ion mode at 22.02 min (Figure 7) showed a deprotonated molecular ion [M − H]^−^ at *m*/*z* 545.3325, from which the molecular formula C_28_H_49_O_10_ (mass error −1.2 ppm) was determined. Furthermore, the mass spectrum showed two in-source fragment ions, corresponding to the fatty acids of the glucose–lipid, namely 3-hydroxy-5-dodecenoic acid (C12:1) and 3-hydroxydecanoic acid (C10:0). These intense fragment ions were also observed in the negative ion mode MS/MS spectrum of *m*/*z* 545.3325 (Figure S13). The MS/MS spectrum of the glucose–lipid was also analyzed by in silico fragmentation of the glucose–lipid structure using the fragment ion search (FISh) tool in the Compound Discoverer software version 3.3, which resulted in excellent agreement between the in silico and acquired MS/MS spectra (Figure S13). Thus, the structure of the glucose–lipid determined by NMR analysis was fully confirmed by the molecular formula (C_28_H_50_O_10_) and the MS/MS spectrum of the glucose–lipid determined by high-resolution LC–ESI–MS/MS.

The NMR- and LC–ESI–MS/MS-based structure elucidation of the glycolipid produced by *R. badensis* DSM 100043^T^ in this recent study filled in the knowledge gaps from the previous study, in which (1) exact carbohydrate moieties, (2) fatty acid moieties, (3) acylation sites, and (4) linkages at the acylation sites had not yet been determined [7]. It was clear that *R. badensis* DSM 100043^T^ produced a structurally novel glycolipid with a single glucose molecule as the hydrophilic moiety and two hydroxy fatty acids, 3-hydroxy-5-dodecenoic acid (C12:1) and 3-hydroxydecanoic acid (C10:0), as the hydrophobic moieties that are linked to ester bonds at the glucose’s acylation sites C2 and C3. LC–ESI–MS/MS and NMR analyses did not detect congeners of glucose–lipid containing only a single fatty acid moiety in subsequent MPLC fractions. The only structural peculiarity of this glucose–lipid was the unclear absence of acylation at the anomeric carbon C1, which is typically found in most glycolipid biosurfactants, such as rhamnolipids and sophorolipids [19,20]. This unique structural property might have been clearly explained if the biosynthesis pathway of this glucose–lipid had been elucidated.

### 2.3. Characterization of Novel Glucose–Lipid Biosurfactant

**Figure 8:**
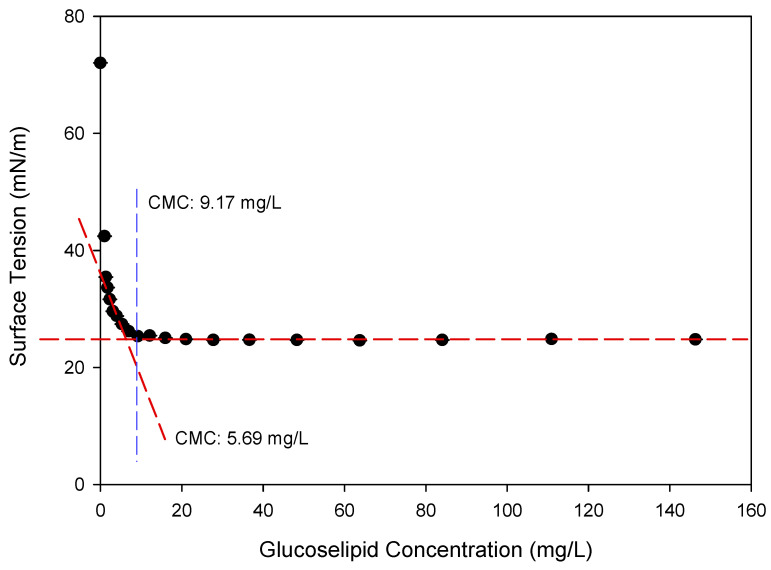
Surface tension curve and CMC determination of glucose–lipid biosurfactant from *R. badensis* DSM 100043^T^. The CMC of 5.69 mg/L was obtained with the tangent-line method (red dashed line), while the CMC of 9.17 mg/L was obtained with the vertical-line method (blue dashed line).

CMC, as one of the crucial parameters of any surface-active chemical, is commonly used to define the surfactant’s efficiency and is one of the variables able to determine the quality of biosurfactant [21]. This parameter corresponds to the lowest concentration of surfactant necessary to attain the minimum surface tension and formation of micelles [22]. Once the CMC values are achieved, adding more surfactants does not lead to any additional decrease in surface tension. Therefore, surfactants with lower CMC values are considered more beneficial for practical applications than those with higher CMC values [23]. As shown in Figure 8, the purified glucose–lipid of *R. badensis* DSM 100043^T^ could reduce the surface tension of water at 25 °C from 72.05 mN/m to 24.59 mN/m. The calculated CMC value as the intersection between the lower horizontal portion of the curve and the tangent line, as exemplified by Wu et al. (2022) and Zhong et al. (2008), was found to be 5.69 mg/L [24,25], while the calculated CMC value as the first concentration point at which surface tension did not decrease anymore, based on experimental data, was 9.17 mg/L [26,27]. These results show that the glucose–lipid produced by *R. badensis* DSM 100043^T^ had excellent surface activity properties, as indicated by the very low CMC value compared to other microbial biosurfactants, as shown in Table 3. This outstanding property might be explained by the structure of the glucose–lipid, where the polar glucose molecule acts as a hydrophilic moiety while the two non-polar fatty acid tails act as a hydrophobic moiety. Moreover, differences in the specific chemical composition, such as the type and length of fatty acid chains, the position and type of glycosidic linkage, and the overall molecular conformation, might also play critical roles in influencing surface activity and CMC values.

**Figure 9:**
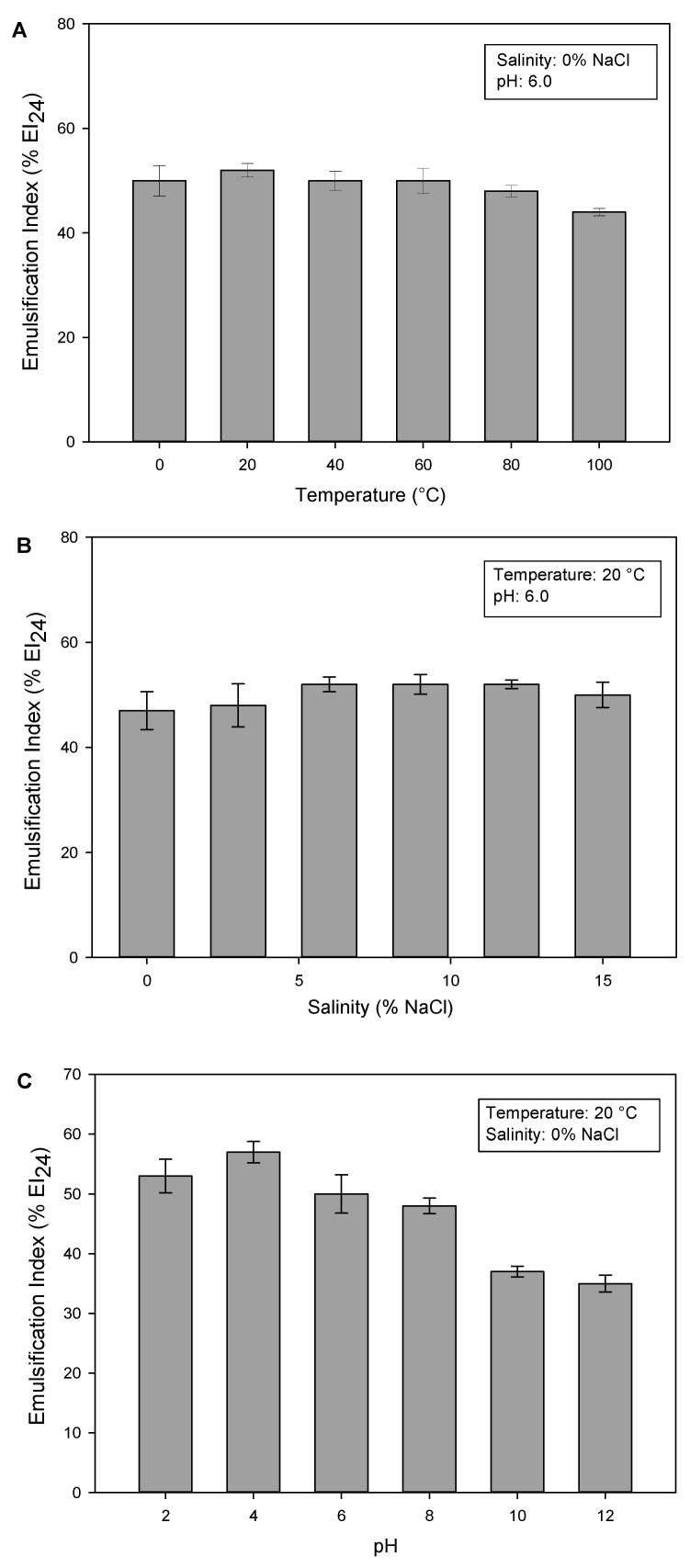
Stability test of 50 mg/L glucose–lipid biosurfactant from *R. badensis* DSM 100043^T^ by measuring the emulsification index in olive oil at different temperature (**A**), salinity (**B**), and pH (**C**).

The stability of biosurfactants under various environmental conditions is a crucial aspect for their utilization across diverse sectors. Marchant and Banat (2012) also asserted that biosurfactant stability is critical for the feasibility of mass production, particularly when employing biotechnological processes for its manufacture [42]. In this study, the stability of the glucose–lipid biosurfactant produced by *R. badensis* DSM 100043^T^ at a different range of temperatures, pH, and salinity was evaluated using an emulsification test (%EI_24_) in olive oil. In the emulsification test, 50 mg/L of pure glucose–lipid biosurfactant was used, which was above its CMC value, to maintain the emulsification performance of the glucose–lipid. In general, the glucose–lipid showed good emulsification stability against changes in slightly acidic pH, temperature, and saline concentration, as shown in Figure 9.

Figure 9A shows the effect of different temperatures on the emulsification index of glucose–lipid in olive oil. The glucose–lipid demonstrated thermostability property, maintaining its stability at temperatures of up to 100 °C. The thermal stability of the glucose–lipid biosurfactant suggests its potential for use across various industries that operate under high temperatures, as well as its suitability for enhanced oil recovery [43]. The glucose–lipid was also found to be stable over a wide range of NaCl concentrations, as shown in Figure 9B. The stability of the glucose–lipid biosurfactant over high salinity demonstrates its potential for the treatment of oil spills in the marine environment [44]. Regarding pH stability, the results showed that pH plays a significant role in the chemical stability of the glucose–lipid biosurfactant, as %EI_24_ changed over different pH values, as shown in Figure 9C. The glucose–lipid was found to be more stable at an acidic pH rather than in a basic environment, where the highest extent of the emulsification index was observed at pH 4. This phenomenon contrasts with that observed in rhamnolipids, where an acidic pH leads to the precipitation of rhamnolipid, thereby reducing its stability [21]. The glucose–lipid biosurfactant of *R. badensis* DSM 100043^T^ did not have an acidic group that could be protonated, meaning that a low pH value should not play a major role in the precipitation, and should remain stable at low pH levels, making it suitable for cosmetic applications where effectiveness is expected on the slightly acidic pH of human skin.

## 3. Materials and Methods

### 3.1. Media and Cultivation Procedures

#### 3.1.1. Shake Flask Cultivations

A modified MSM based on the fermentation medium of Kügler et al. (2015) was used for cultivation experiments in shake flasks [7]. The initial pH of the medium was set to 7.0 by 4 M NaOH, and it consisted of 4.4 g/L K_2_HPO_4_, 3.4 g/L KH_2_PO_4_, 3.0 g/L (NH_4_)_2_SO_4_, 1.1 g/L NaCl, 1.1 g/L KCl, and 0.0008 M MgSO_4_·7H_2_O. The medium was supplemented with 5 mL of trace element solution containing 0.29 g ZnSO_4_·7H_2_O, 0.14 g CaCl_2_, 0.47 g FeSO_4_·7H_2_O, 0.17 g MnSO_4_·H_2_O, 0.25 g CuSO_4_·5H_2_O, and 0.11 g EDTA disodium salt dihydrate per liter.

The first preculture was prepared by inoculating 25 μL *R. badensis* DSM 100043^T^ glycerol stock in 25 mL LB medium (10 g/L tryptone,5 g/L NaCl, 5 g/L yeast extract) in 250 mL baffled shake flasks. The first preculture was incubated in an incubator shaker (120 rpm, 12 h, 30 °C, Innova 44^®^R Eppendorf AG, Hamburg, Germany) and was used to prepare the second preculture with a starting OD_600_ of 0.1. The second preculture was prepared in 100 mL MSM supplemented with 25 g/L glycerol in a 1 L baffled shake flask and was incubated under the same conditions for 12 h.

The main culture was performed in 100 mL MSM in a 1 L baffled shake flask with a starting OD_600_ of 0.1. The first part of the shake flask experiments aimed to investigate the effects of incubation temperature (20–30 °C) on cell growth and glucose–lipid production at 15 g/L glycerol as a carbon source, while the second part aimed to investigate the effects of glycerol concentration (10–30 g/L) at an incubation temperature of 30 °C. All cultivations were carried out in biological triplicate in incubator shakers (120 rpm, 32 h, Innova 44^®^R Eppendorf AG, Hamburg, Germany). Samples were taken at regular intervals and the OD_600_ was measured using a spectrophotometer (WPA CO8000, Biochrom Ltd., Cambridge, UK), then subsequently centrifuged (4700 rpm, 10 min, 4 °C, Heraeus Multifuge X3R, Thermo Fisher Scientific, Waltham, MA, USA) to remove cell pellets. The supernatants were stored at −20 °C for further analysis.

#### 3.1.2. Bioreactor Cultivation

The first and second precultures for bioreactor cultivations of *R. badensis* DSM 100043^T^ were prepared in the same manner for the shake flask cultivations mentioned above. Minor changes were applied for the second preculture, which was performed in a final working volume of 200 mL in a 2 L baffled flask equipped with an outlet for inoculation. The medium used for bioreactor cultivation was an identical MSM used for the shake flask cultivations, supplemented with 15 g/L glycerol as the carbon source. The required inoculation volume to start with an initial OD_600_ of 0.3 was then calculated and pumped into the bioreactor.

Bioreactor cultivation was carried out in batch mode using a 10 L glass bioreactor system (Satrorius, BIOSTAT^®^ Bplus, Sartorius Stedim Systems GmbH, Göttingen, Germany) with a filling volume of 6 L. The bioreactor was equipped with a pH sensor (EasyFerm Bio HB K8 325, Hamilton Bonaduz AG, Bonaduz, Switzerland) and a pO_2_ sensor (VisiFerm DO ARC 120 H0, Hamilton Bonaduz AG, Bonaduz, Switzerland), a temperature sensor, and two Rushton turbines. The temperature of the bioreactor was set to 30 °C and the pH was controlled at 7.0 by using 4 M NaOH and 4 M H_3_PO_4_. Before inoculation, 600 μL antifoam (STRUKTOL^®^ SB590, Schill + Seilacher “Struktol” GmbH, Hamburg, Germany) was added to the medium to prevent excessive foam formation. At the beginning of fermentation, the air flowrate was set to a constant 1.0 L/min. The agitation rate and air flowrate were then regulated by online control to maintain dissolved oxygen at a minimum level of 20%.

Samples were taken at regular intervals and OD_600_ was measured using a spectrophotometer (WPA CO8000, Biochrom Ltf., Cambridge, UK), then subsequently centrifuged (4700 rpm, 10 min, 4 °C, Heraeus Multifuge X3R) to remove cell pellets. The supernatants were stored at −20 °C for further analysis.

### 3.2. Sample Quantitative Analysis

#### 3.2.1. Glycerol and Ammonia Quantification

Glycerol quantification in the supernatant was carried out using an enzymatic assay kit following the protocol of the manufacturer (K-GCROL, Megazyme, Wicklow, Ireland). Ammonia quantification in the supernatant was carried out using an enzymatic assay kit following the protocol of the manufacturer (Art. No. 11112732035, R-Biopharm AG, Pfungstadt, Germany). The adsorption shifts at 340 nm were measured with a UV-visible spectrophotometer (GENESYS 150, Thermo Scientific, Waltham, MA, USA). Standard as well as blank measurements were completed regularly each time when performing the assay, if not stated otherwise.

#### 3.2.2. Glucose–Lipid Quantification

Glucose–lipid was initially precipitated from 2 mL supernatant by the addition of 20 μL 85% *v*/*v* H_3_PO_4_. The acidification was followed by two-fold liquid–liquid extraction, where 2.5 mL ethyl acetate was added to the acidified supernatant and mixed for 5 s. After centrifugation (3000 rpm, 15 min, 4 °C, Heraeus Multifuge X3R), 1.5 mL of the upper phase was transferred to a 15 mL falcon tube, resulting in a final volume of 3 mL. To concentrate the glucose–lipid, the sample was evaporated at 10 mbar and 40 °C for 40 min using the rotavapor (RVC 2-25 CDplus, Martin Christ, Osterode am Harz, Germany). The residue was resolved in 1 mL ethyl acetate for subsequent high-performance thin-layer chromatography (HPTLC) measurement.

The glucose–lipid quantification method using HPTLC was first developed and then validated based on Validation of Analytical Procedures: Methodology (FDA Guidance) with respect to the parameters of linearity, limit of detection (LOD), limit of quantification (LOQ), precision, accuracy, and repeatability, according to the Food and Drug Administration (FDA) Guidance (1999) [9,45]. Purified glucose–lipid (95% purity) was dissolved in ethyl acetate, then used as a reference material at a concentration of 1 g/L. Calibration curves were developed by applying different volumes (5; 10; 15; 20; 25; 30; 35; 40 μL) of this reference material on a thin-layer chromatography (TLC) plate, then the glucose–lipid concentration in the samples was calculated by considering the 95% purity of the reference material.

WinCATS Software 1.4.7 (CAMAG, Muttenz, Switzerland) was used to control all the HPTLC instruments (CAMAG, Muttenz, Switzerland). A sufficient amount of sample material was applied automatically by an Automatic TLC Sampler 4 (ATS 4) on 10 × 20 cm TLC silica gel 60 RP-18 F_254S_ (Merck KGaA, Darmstadt, Germany) 8 mm away from the lower edge and 15 mm away from the left edge. The application parameters were set as follows: filling speed 15 μL/s, dosage speed 150 nL/s, filling vacuum time 1.0 s, rinsing vacuum time 2.0 s, band length 6.0 mm. Methanol was used as the rinsing solvent. The development was performed using an automatic developing chamber (ADC 2) equipped with a 20 cm × 10 cm twin-trough chamber using 10 mL isopropyl acetate/methanol/acetic acid (100:10:1, *v*/*v*/*v*) as the mobile phase until a migration distance of 70 mm was reached. A chamber saturation step for 5 min was attained to reach equilibrium between the vapor and the solvent. The preconditioning step was carried out for 1 min by using a filter paper soaked with 25 mL of the mobile phase. A final drying step was then performed for 5 min. DPA was used as a derivatization reagent to visualize the glucose–lipid on the TLC plate, as DPA has been found to be sensitive, convenient, and widely used for revealing glycoconjugates on TLC plates [46,47,48,49]. The DPA reagent was prepared by dissolving 2.4 g diphenylamine and 2.4 g aniline in 200 mL methanol, then adding 20 mL 85% H_3_PO_4_. The developed plate was immersed in the derivatization reagent using a TLC immersion device at 3 cm/s speed for 3 s, and the reaction was completed by incubating the plate on a TLC Plate Heater 3 at 120 °C for 10 min. The derivatized plate was scanned with a scanning speed of 20 mm/s, a data resolution of 100 μm/step, and a slit dimension of 3.0 × 0.30 mm at 620 nm by a TLC Scanner 4. For glucose–lipid quantification, peak areas were evaluated using a linear regression based on a calibration curve obtained from the reference material.

#### 3.2.3. Emulsification Assay

An emulsification assay was carried out as an indirect method of observing glucose–lipid production and performance as a biosurfactant in bioreactor cultivation. Three milliliters of cell-free supernatant samples were mixed with 0.5 mL test oil and vortexed vigorously for 2 min, then incubated at 37 °C for 1 h. In this assay, both commercial olive oil (P. Brändle GmbH, Empfingen, Germany) and low-viscosity paraffin oil (Carl Roth, Karlsruhe, Germany) were used as test oils. The aqueous phase was transferred to a cuvette and the absorbance was recorded at 400 nm using a UV-visible spectrophotometer (GENESYS 150, Thermo Scientific, Waltham, MA, USA). The blank was prepared similarly with sterile MSM for bioreactor cultivation. At 400 nm, an absorbance of 0.010 units multiplied by any applicable dilution factor is considered equivalent to one unit of emulsification activity per milliliter (EU/mL) [50].

### 3.3. Structure Elucidation of Glycolipid

#### 3.3.1. Isolation and Purification of Glucose–Lipid

Fermentation broth from bioreactor cultivation was first subjected to centrifugation at 4700 rpm and 4 °C for 15 min (Heraeus Multifuge X3R) to separate the supernatant from the cell pellets. The cell-free supernatant was then acidified with 1% volume 85% H_3_PO_4_ (*v*/*v*) and subsequently extracted twice using 1.25 volumes of ethyl acetate (*v*/*v*) in a separation funnel. The extraction process was carried out by manually shaking the separation funnel vigorously for 5 min at room temperature. The organic phase was taken out and concentrated using a rotary vacuum evaporator (R-215, Büchi Labortechnik AG, Flawil, Switzerland) at 215 mbar and 40 °C, followed by further vacuum evaporation with a rotavapor (RVC 2-25 CDplus, Martin Christ, Osterode am Harz, Germany) at 10 mbar, 1200 rpm, and 40 °C for 6 h to obtain the final crude extract.

The crude extract was dissolved in 3 mL dimethyl sulfoxide (DMSO) for purification with medium-pressure liquid chromatography (MPLC; SepacoreX50, Büchi, Flawil, Switzerland) using a prepacked 40–60 μm particle size reverse phase C18 column (FlashPure EcoFlex C18; 40 g; column volume 80 mL, Büchi, Flawil, Switzerland). In this recent study, a 7.5 mL/min acetonitrile (ACN)/water gradient system was used as the mobile phase, replacing the previous method, which used a 10 mL/min water/methanol gradient system to achieve better separation [7]. The reverse-phase C18 column with an ACN/water gradient (30–70% to 70–100% ACN) and the addition of acids or ammonium acetate is very common and the most reliable method for the purification of rhamnolipids as one of the most well-known glycolipid biosurfactants [51,52]. Moreover, this method was also used for the purification of surfactin, a notable lipopeptide biosurfactant, from 50% to 90% ACN in a gradient system [53,54]. The chromatography step was run for 120 min (gradient: 10 min 45–45% ACN; 90 min 45–100% ACN; 10 min 100–100% ACN; 10 min 100–50% ACN). The eluate was collected in 10 mL fractions. All fractions were then analyzed by using HPTLC, where silica gel 60 RP-18 F_254S_ plates were derivatized with p-anisaldehyde/sulfuric acid/glacial acetic acid (1:2:100 *v*/*v*/*v*) reagent. Fractions containing the glucose–lipid were pooled, and solvents were evaporated by using a rotary vacuum evaporator (R-215, Büchi Labortechnik AG, Switzerland) at 10 mbar and 40 °C for structure elucidation.

#### 3.3.2. Nuclear Magnetic Resonance (NMR) Analysis

For the structure elucidation of the glycolipid, 1D and 2D NMR spectra were recorded on an Avance HD III 600 MHz spectrometer equipped with a 5 mm BBO Prodigy cryo-probe (Bruker, Billerica, MA, USA). The sample was dissolved in 600 μL methanol-*d*4 and transferred to a standard 5 mm NMR tube. ^1^H and ^13^C chemical shifts were referenced to the residual solvent signal at δ_H/C_ 3.35 ppm/ 49.0 ppm. ^1^H, ^13^C, HSQC, HMBC, COSY, heteronuclear single-quantum coherence total correlation spectroscopy (HSQCTOCSY), F1 homoband-decoupled HSQC, band-selective HSQC with and without decoupling, band-selective HMBC, heteronuclear 2-bond correlation (H2BC), and selective 1D-TOCSY spectra were recorded using standard Bruker pulse sequences at 298 K. A super-long-range HMBC was measured by an in-house-modified Bruker pulse sequence. Triple-spin echo pure shift yielded by chirp excitation (TSE-PSYCHE) and F1-homodecoupled PSYCHE TOCSY pulse sequence and parameters were obtained from the Manchester NMR methodology group [13,14,15]. The recorded NMR spectra were processed with Topspin 4.1.3 (copyright 2021, Bruker Biospin, Billerica, MA, USA) and SpinWorks 4.2.10 (Copyright 2019, K. Marat, University of Manitoba, Winnipeg, MB, Canada).

#### 3.3.3. Liquid Chromatography Electrospray Ionization Mass Spectrometry (LC-ESI/MS)

LC–ESI–MS/MS analysis of the glycolipid was performed on a 1290 ultra-high-performance liquid chromatography (UHPLC) system (Agilent, Waldbronn, Germany) coupled to a Q-Exactive Plus Orbitrap mass spectrometer, as previously described in Vahidinasab et al. (2022), with modifications [55]. In summary, the separation of glycolipids was accomplished using an ACQUITY CSH C18 column (1.7 μm, 2.1 μm × 150 mm, Waters, Eschborn, Germany). The mobile phase A was composed of 0.2% formic acid in water, while mobile phase B consisted of 0.2% formic acid in methanol. Gradient elution was executed using a constant flow rate of 0.3 mL/min, with the following percentage ranges of mobile phase B: 10–25% from 0 to 5 min, 25–45% from 5 to 10 min, 45–90% from 10 to 20 min, 90% isocratic from 20 to 21 min, and 10% from 21 to 26 min. Subsequently, the system was returned to its initial conditions at 90% from 26 to 21 min and re-equilibrated at 10% from 26 to 31 min.

The HESI source was operated in positive and negative ion modes, with a spray voltage of 4.0 kV in positive ion mode and 3.5 kV in negative ion mode. Mass spectra were acquired in the mass range of 100 to 1400 *m*/*z* at a resolution of 70,000 FWHM. Data-dependent MS/MS spectra in the mass range of 50 to 2000 *m*/*z* were generated for the five most abundant precursor ions at a resolution of 17,500 FWHM and a normalized collision energy of 19. The data acquisition and analysis were performed using Xcalibur software version 4.3.73.11 and Compound Discoverer Software version 3.3 (both Thermo Fisher Scientific, San Jose, CA, USA). The identification and assignment of the glucose–lipid were based on the precise *m*/*z* value of the precursor ion and manual inspection of the corresponding MS/MS spectra. The structure predictions derived from nuclear magnetic resonance (NMR) were then compared to the MS/MS spectra, utilizing in silico fragmentation prediction provided by the fragment ion search (FISh)/mass frontier algorithm in Compound Discoverer.

### 3.4. Characterization of Glucose–Lipid

#### 3.4.1. Critical Micelle Concentration (CMC) Determination

CMC was determined by measuring the surface tension of the air–water surface at 25 °C using a DCAT 11 tensiometer equipped with a Wilhelmy plate (DataPhysics GmbH, Filderstadt, Germany). The glass beaker and Wilhelmy plate were cleaned with purified water and ethanol and then rinsed thoroughly with purified water. The Wilhelmy plate was further heated to a light red glow with a Bunsen burner to remove any contaminants. The stock aqueous glucose–lipid solution (800 mg/L, M = 546 g mol^−1^) was titrated manually into purified water in a glass beaker in a series of increasing volumes. After each titration, the sample solution was stirred at a 30% stirring rate for 60 s and equilibrated for 10 s before measuring the surface tension. The surface tension of the purified water was 72.049 ± 0.012 mN m^−1^. The density of the glucose–lipid solution was 0.99780 kg m^−3^, as determined by using a DMA 35N density meter (Anton Paar GmbH, Graz, Austria).

#### 3.4.2. Stability Test

The stability of the glucose–lipid biosurfactant was evaluated under a wide range of temperature, salinity, and pH above its CMC. A modified method from Samykannu et al. (2017) was performed using 2 mL of 50 mg/L glucose–lipid dissolved in ultrapure water [43]. To analyze the temperature stability, the glucose–lipid solution was incubated at 0, 20, 40, 60, 80, and 100 °C for 1 h. To analyze the stability on different salinities, the glucose–lipid solution was incubated at 0, 3, 6, 9, 12, and 15% (*w*/*v*) NaCl. To examine the pH stability of the glucose–lipid biosurfactant, the pH of the glucose–lipid solution was adjusted to different pH values ranging from 2 to 12 using 6 N HCl and 6 N NaOH. All samples were then subjected to an emulsification activity test (E_24_) based on Hamzah et al. (2020), where 2 mL of olive oil was added to incubated samples, vortexed for 5 min, and incubated again for 24 h [56]. The E_24_ index was obtained by calculating the percentage of the height of the emulsified layer divided by the total height of the liquid column [57].

### 3.5. Data Analysis

The formulas presented in Hoffmann et al. (2020) were used to calculate growth rate (*μ*), yield cell per substrate (*Y*_X/S_), yield product per substrate (*Y*_P/S_), yield product per cell (*Y*_P/X_), and specific productivity (*q*_P/X_) [58]. Parameter *Y*_X/S_ was calculated at the maximum cell dry weight (CDW_max_), while *Y*_P/S_, *Y*_P/X_, and *q*_P/X_ were calculated at the maximum glucose–lipid concentration during cultivation [55]. The fitting curves shown in all figures were derived using scientific graphing and data analysis software (SigmaPlot 15.0, Systat Software Inc., San Jose, CA, USA).

## 4. Conclusions

This study investigated the structure elucidation of a glycolipid biosurfactant produced by *R. badensis* strain DSM 100043^T^. The optimal growth conditions to obtain the highest glucose–lipid concentration in shake flask at 30 °C and 15 g/L glycerol were applied in bioreactor cultivation. Prior to structure elucidation, the purification of ethyl acetate–crude extract using MPLC equipped with a reverse-phase hydrophobic C18 column and an ACN/water gradient system was successfully performed to obtain pure glucose–lipid fractions. Using two-dimensional NMR spectroscopy, the hydrophilic moiety of *R. badensis*’s glycolipid was elucidated as glucose. On the other hand, the hydrophobic moieties were revealed as 3-hydroxy-5-dodecenoic acid (C12:1) and 3-hydroxydecanoic acid (C10:0), both of which are linked to acylation sites C2 and C3 of glucose via ester bonds. The NMR results were truly confirmed by negative ion mode LC–ESI/MS, where the glucose–lipid molecule eluted at a retention time of 22.2 min. Calculation based on the deprotonated glucose–lipid molecule [M − H]^−^ with *m*/*z* value of 545.33312 indicated that the sum formula of the neutral molecule was C_28_H_50_O_10_ and was in good agreement with the NMR results. The glucose–lipid produced by *R. badensis* DSM 100043^T^ obtained in this study was identified as a novel compound, thus expanding our knowledge for broader types of microbial glycolipid biosurfactants beyond well-known substances such as rhamnolipids, sophorolipids, and mannosylerythrithol lipids (MELs). Remarkably, CMC determination showed that the glucose–lipid had excellent surface-active properties, as indicated by the very low CMC and minimum surface tension values compared to other microbial glycolipid biosurfactants. Moreover, the stability test showed that the glucose–lipid had high stability at a wide range of temperatures, salinity, and relatively low pH levels.

## Figures and Tables

**Table 1:** Overview of process parameters for shake flask and bioreactor cultivations of *R. badensis* DSM 100043^T^. Complete growth parameters showing *Y*_X/S_, *Y*_P/S_, and *Y*_P/X_ can be found in Table S3.

Parameter	Shake Flasks (15 g/L Glycerol)			Shake Flasks (30 °C)			Bioreactor
	20 °C	25 °C	30 °C	10 g/L	20 g/L	30 g/L	
CDW_max_ (g/L)	6.26	6.55	5.41	4.98	6.12	5.48	5.84
*μ* (1/h)	0.25	0.32	0.34	0.34	0.31	0.29	0.34
*μ*_max_ (1/h)	0.32 (14 h)	0.43 (14 h)	0.53 (12 h)	0.52 (12 h)	0.54 (12 h)	0.48 (12 h)	0.48 (8 h)
*P*_max_ (mg/L)	69.9 (32 h)	60.3 (28 h)	89.3 (28 h)	31.7 (32 h)	55.9 (28 h)	49.8 (32 h)	42.9 (28 h)
*q*_P/X_ (g/(g·h))	0.00043	0.00038	0.00066	0.00023	0.00036	0.00029	0.00031

**Table 2:** ^1^H and ^13^C NMR chemical shifts, spin multiplicity, and J coupling constants of the α- and β-anomeric form of glucose–lipid sample in methanol-d4 at 600 (^1^H) and 150 MHz (^13^C).

Atom Number	α-Anomer		β-Anomer	
	δ_H_ (ppm), Multiplicity, *J* (Hz)	δ_C_ (ppm)	δ_H_ (ppm), Multiplicity, *J* (Hz)	δ_C_ (ppm)
1	5.34, d, 3.6	91.19	4.76, d, 8.0 ^a^	96.15
2	4.78, dd, 3.6, 10.2	73.35	4.81, dd, 8.0, 9.6	74.53
3	5.48, dd, 9.4, 10.1	73.99	5.11, t-like, 9.4	77.00
4	3.64, t-like, 9.7	69.72	3.59, t-like, 9.6 ^a^	69.72
5	3.93, ddd, 2.3, 4.7, 10.0	72.78	3.43, ddd, 2.0, 5.3, 9.8	77.96
6a/b	3.84, dd, 2.4, 12.0 3.78, dd, 4.8, 11.9	62.17	3.88, dd, 2.2, 12.0 ^a^ 3.72, dd, 5.5, 12.0 ^a^	62.40
1′	-	172.65	-	172.30
2′	2.57, dd, 8.8, 15.3 2.41, dd, 4.2, 15.3	42.66	2,59, dd, 4.2,15.2 2.39, ov	42.73
3′	4.02, m	69.35	4.02, ov	69.22 ^b^
4′	2.28, br t, 6.9	35.93	2.29, ov	35.83
5′	5.45, dtt, 1.5, 7.3, 10.9	125.95	5.45, ov	126.00
6′	5.54, dtt, 1.5, 7.4, 10.9	133.61	5.54, ov	133.59
7′	2.10, br q-like, 7.1	28.42	2.10, ov ^c^	28.42, ov ^c^
8′	1.39, quintet, 7.2 ^d^	30.73	1.39, ov ^c^	30.73, ov ^e^
9′	1.36, quintet, 7.9 ^d^	30.12	1.36, ov ^c^	30.12, ov ^c^
10′	1.31, quintet, 7.9 ^d^	33.04 ^f^	1.31, ov ^c^	33.04 ^f^, ov ^c^
11′	1.34, sextet, 7.4 ^d^	23.72 ^g^	1.34, ov ^c^	23.72 ^g,^ ov ^c^
12′	0.93, t, 6.7	14.45	0.95, ov ^c^	14.45, ov ^c^
1″	-	173.06	-	172.99
2″	2.57, dd, 4.3, 15.1 2.47, dd, 8.4, 15.1	43.49	2.59, ov 2.47, ov	43.42
3″	4.01, m	69.26	4.01, ov ^c^	69.19 ^b^
4″	1.51, m ^d^	38.17	1.51, ov ^c^	38.17, ov ^c^
5″	1.47, m ^d^ 1.39, m ^d^	26.69	1.47, ov ^c^ 1.39, ov ^c^	26.67
6″	1.34, ov	30.71	1.34, ov ^c^	30.69 ^e^
7″	1.34, quintet, 8.0 ^d^	30.48	1.34, ov ^c^	30.48, ov ^c^
8″	1.31, quintet, 7.9 ^d^	32.95 ^f^	1.31, ov ^c^	32.95 ^f^, ov ^c^
9″	1.34, sextet, 7.4 ^d^	23.74 ^g^	1.34, ov ^c^	23.74 ^g^, ov ^c^
10″	0.93, t, 6.7	14.45	0.95, ov ^c^	14.45, ov ^c^

ov: overlapped by other signals; br: broad; ^a^ coupling pattern and constants derived by selective 1D TOCSY; ^b,e,f,g^ maybe interchanged; ^c 1^H and ^13^C NMR signals completely overlapped and therefore tentatively assigned; ^d^ coupling pattern and constants derived by band-selective HSQC without decoupling. Coupling constants were directly taken from the spectra and are not averaged.

**Table 3:** Comparison of surface tension and CMC values of different microbial biosurfactants.

Surfactant Type	Microorganism	Minimum Surface Tension (mN/m)	CMC (mg/L)	Reference
Glucose–lipid	*Rouxiella badensis* DSM 100043^T^	24.59	5.69	This study
Rhamnolipid	*Pseudomonas aeruginosa* ATCC 9027	34.01	180	[28]
	*Pseudomonas aeruginosa* PTCC 1340	25.86	90	[29]
	*Pseudomonas aeruginosa* R4	32.5	50	[30]
Sophorolipid	*Starmerella bombicola NRRL* Y-17069	34.15	59.43	[31]
	*Rhodotorula babjevae* YS3	32.6	130	[32]
	*Candida bombicola* ATCC 22214	48	150	[33]
MELs *	*Sporisorium* sp. aff. *sorghi* SAM20	30	20	[34]
	*Pseudozyma aphidis* ZJUDM34	30.63	20	[35]
	*Candida antarctica* SY16	29	10	[36]
Trehalolipid	*Rhodococcus* sp., PML026	29	250	[37]
	*Rhodococcus erythropolis* S67	27	32	[38]
Surfactin	*Bacillus nealsonii* S2MT	34.5	40	[39]
	*Bacillus amyloliquefaciens* C11	28	51.5	[40]
	*Bacillus subtilis Isolate* BS5	36	15.6	[41]

* MELs: mannosylerythritol lipids.

## Data Availability

The data that support the findings of this study are available in the Supplementary Materials or can be provided by the corresponding author upon reasonable request.

## Supplementary Materials

The following supporting information can be downloaded at: https://www.mdpi.com/article/10.3390/molecules30081798/s1, Figure S1: Batch cultivations of *R. badensis* DSM 100043^T^ in shake flasks using different temperatures (a.1,a.2) and glycerol concentrations (b.1–b.3). Cultivations were carried out with 15 g/L glycerol for experiment (a.1,a.2) and at 30 °C for experiment (b.1–b.3). Figure S2: ^1^H NMR of freshly dissolved glucose–lipid (blue) and ^1^H NMR after 24 h (red) in methanol-*d*_4_ at 600 MHz. Figure S3: ^13^C NMR spectrum of the glucose–lipid in methanol-*d*_4_ at 150 MHz. Figure S4: Gradient COSY spectrum of the glucose–lipid in methanol-*d*_4_ at 600 MHz. Figure S5: F1-homodecoupled PSYCHE TOCSY spectrum of the glucose–lipid in methanol-*d*_4_ at 600 MHz. Figure S6: Expansion of the F1 homoband-decoupled HSQC spectrum of the glucose–lipid in methanol-*d*_4_ at 600 MHz displaying the region of carbohydrate resonances (upper) and band-selective HSQC without decoupling to determine the *J*_HH_-coupling constants of strongly overlapped signals (lower). Figure S7: HMBC spectrum of the glucose–lipid in methanol-*d*_4_ at 600 MHz. Figure S8: Band-selective HMBC spectrum of the glucose–lipid in methanol-*d*_4_ at 600 MHz displaying the aliphatic region. Figure S9: H2BC spectrum of the glucose–lipid in methanol-*d*_4_ at 600 MHz displaying the aliphatic region. Figure S10: PSYCHE (upper trace) and ^1^H NMR spectrum (lower trace) of the glucose–lipid in methanol-*d*_4_ at 600 MHz. Figure S11: Expansion of the HSQCTOCSY spectrum of the glucose–lipid in methanol-*d*_4_ at 600 MHz displaying the aliphatic region. Figure S12: Expansion of the band-selective super-long-range HMBC spectrum of the glucose–lipid in methanol-*d*_4_ at 600 MHz displaying ^4^*J*_CH_ of terminal methyl groups of FAs. Figure S13: Fragment ion search (FISh) scoring in the negative ion mode MS/MS spectrum of m/z 545.3325 of the purified glucose–lipid sample. The theoretical fragmentation of the glycolipid structure determined by NMR analysis is in excellent agreement with the acquired MS/MS spectrum of m/z 545.3325 in negative ion mode. Table S1. Determination of the conversion factor between cell dry mass and optical density in bioreactor cultivation (18 h) of *R. badensis* DSM 100043^T^. Table S2. Determination of the conversion factor between cell dry mass and optical density in shake flask cultivation of *R. badensis* DSM 100043^T^. Table S3. Overview of growth parameters showing *Y*_X/S_, *Y*_P/S_, and *Y*_P/X_ for shake flask and bioreactor cultivations of *R. badensis* DSM 100043^T^.
