# Supplementary material for: Structure Elucidation and Characterization of Novel Glycolipid Biosurfactant Produced by Rouxiella badensis DSM 100043T
Source: Molecules. 2025 Apr 17;30(8):1798. doi: 10.3390/molecules30081798 (PMC12029617; doi:10.3390/molecules30081798)
Supplement: Supplementary file 1 [file molecules-30-01798-s001.zip › molecules-3569622-supplementary.pdf]

Supplementary Information SM1

Structure Elucidation and Characterization of Novel Glycolipid Biosurfactant Produced by *Rouxiella badensis* DSM 100043^T

Andre Fahriz Perdana Harahap [1], Jürgen Conrad [2], Mario Wolf [2], Jens Pfannstiel [3], Iris Klaiber [3], Jakob Grether [1], Eric Hiller [1], Maliheh Vahidinasab [1], Hanna Salminen [4], Chantal Treinen [5], Elvio Henrique Benatto Perino [1] and Rudolf Hausmann [1,*]

[1] Department of Bioprocess Engineering (150k), Institute of Food Science and Biotechnology, University of Hohenheim, Fruwirthstr. 12, 70599 Stuttgart, Germany; andrefahrizperdana.harahap@uni-hohenheim.de (A.F.P.H.); jakob.grether@uni-hohenheim.de (J.G.); eric.hiller@uni-hohenheim.de (E.H.); malihe.vahidinasab@uni-hohenheim.de (M.V.); eperino@uni-hohenheim.de (E.H.B.P.)

[2] Department of Organic Chemistry (130b), Institute of Chemistry, University of Hohenheim, Garbenstr. 30, 70599 Stuttgart, Germany; juergen.conrad@uni-hohenheim.de (J.C.); wolf.mario@uni-hohenheim.de (M.W.)

[3] Mass Spectrometry Unit, Core Facility Hohenheim, University of Hohenheim, Ottilie-Zeller-Weg 2, 70599 Stuttgart, Germany; jens.pfannstiel@uni-hohenheim.de (J.P.); iris.klaiber@uni-hohenheim.de (I.K.)

[4] Department of Food Material Science (150g), Institute of Food Science and Biotechnology, University of Hohenheim, Garbenstr. 21/25, 70599 Stuttgart, Germany; hanna.salminen@uni-hohenheim.de

[5] Cellular Agriculture, TUM School of Life Sciences, Technical University of Munich, 85354 Freising, Germany; chantal.treinen@tum.de

* Correspondence: rudolf.hausmann@uni-hohenheim.de

(a.1)

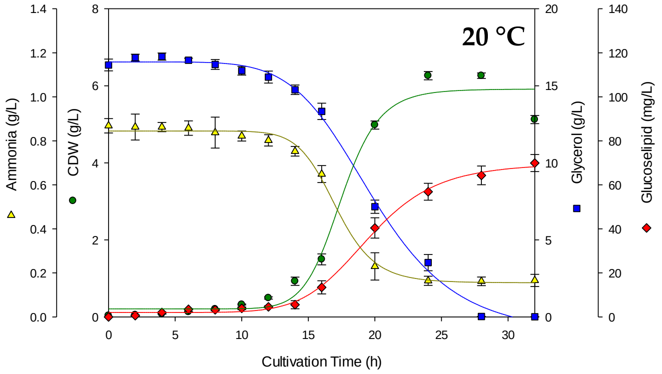
20 °C
Ammonia (g/L)
CDW (g/L)
Glycerol (g/L)
Glucoselipid (mg/L)
Cultivation Time (h)

(b.1)

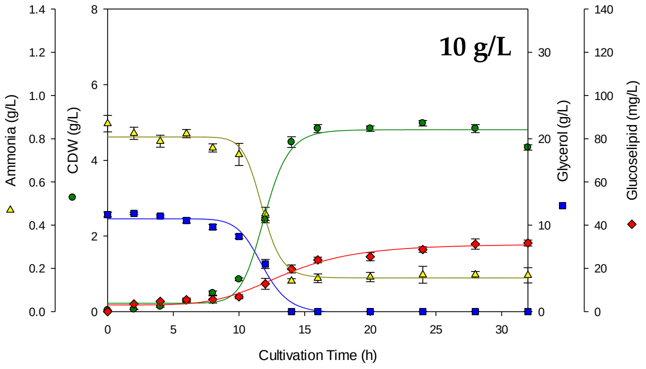
10 g/L
Ammonia (g/L)
CDW (g/L)
Glycerol (g/L)
Glucoselipid (mg/L)
Cultivation Time (h)

(a.2)

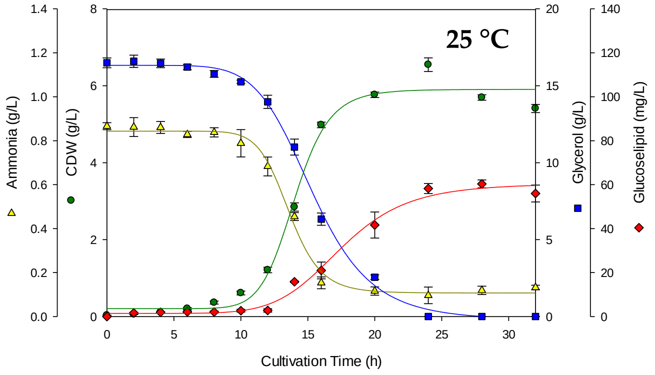
25 °C
Ammonia (g/L)
CDW (g/L)
Glycerol (g/L)
Glucoselipid (mg/L)
Cultivation Time (h)

(b.2)

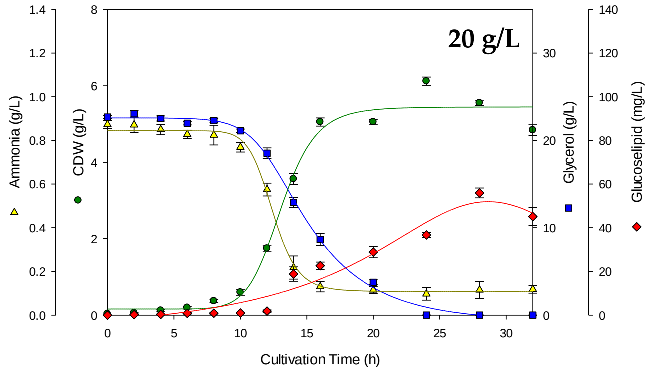
20 g/L
Ammonia (g/L)
CDW (g/L)
Glycerol (g/L)
Glucoselipid (mg/L)
Cultivation Time (h)

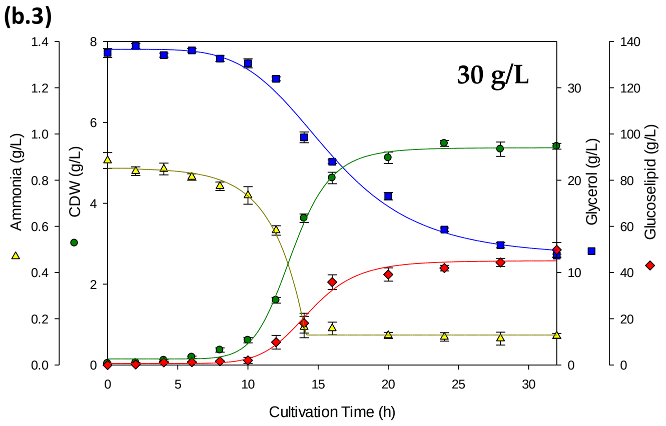

Figure S1 Batch cultivations of *R. badensis* DSM 100043^{T} in shake flasks using different temperatures (**a.1**,**a.2**) and glycerol concentrations (**b.1**–**b.3**). Cultivations were carried out with 15 g/L glycerol for experiment (**a.1**,**a.2**) and at 30 °C for experiment (**b.1**–**b.3**).

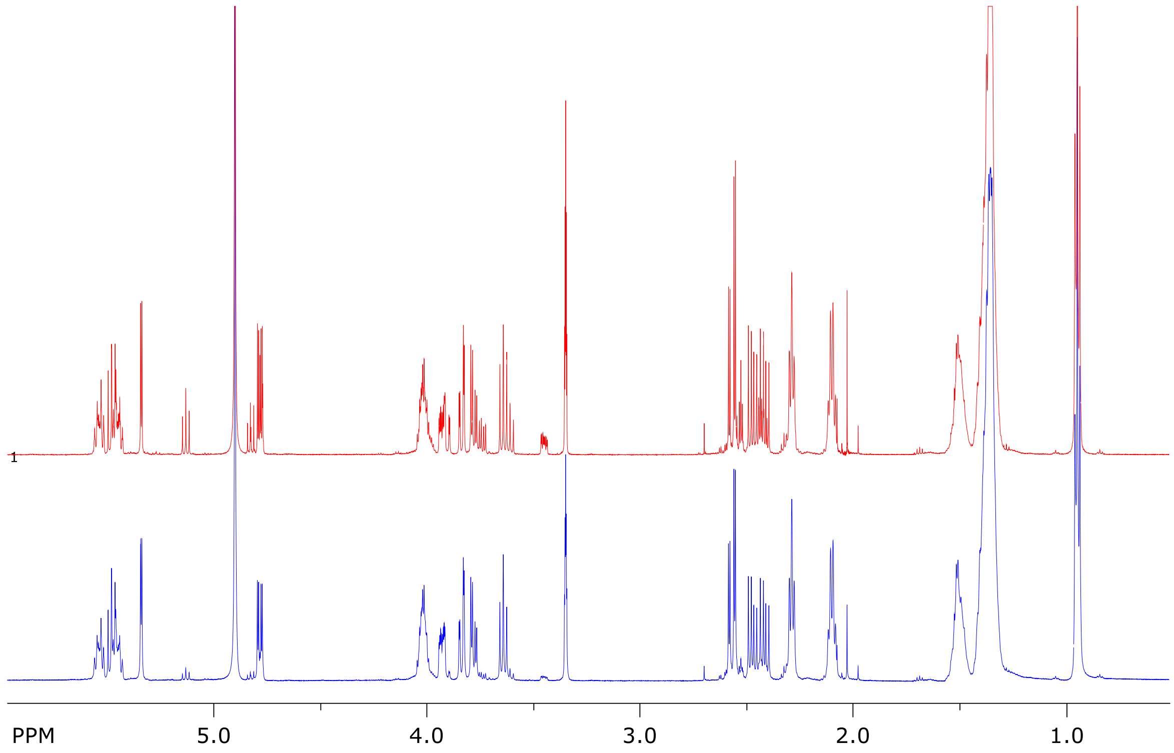

Figure S2 ^{1}H NMR of freshly dissolved glucose–lipid (blue) and ^{1}H NMR after 24 h (red) in methanol-d_4 at 600 MHz.

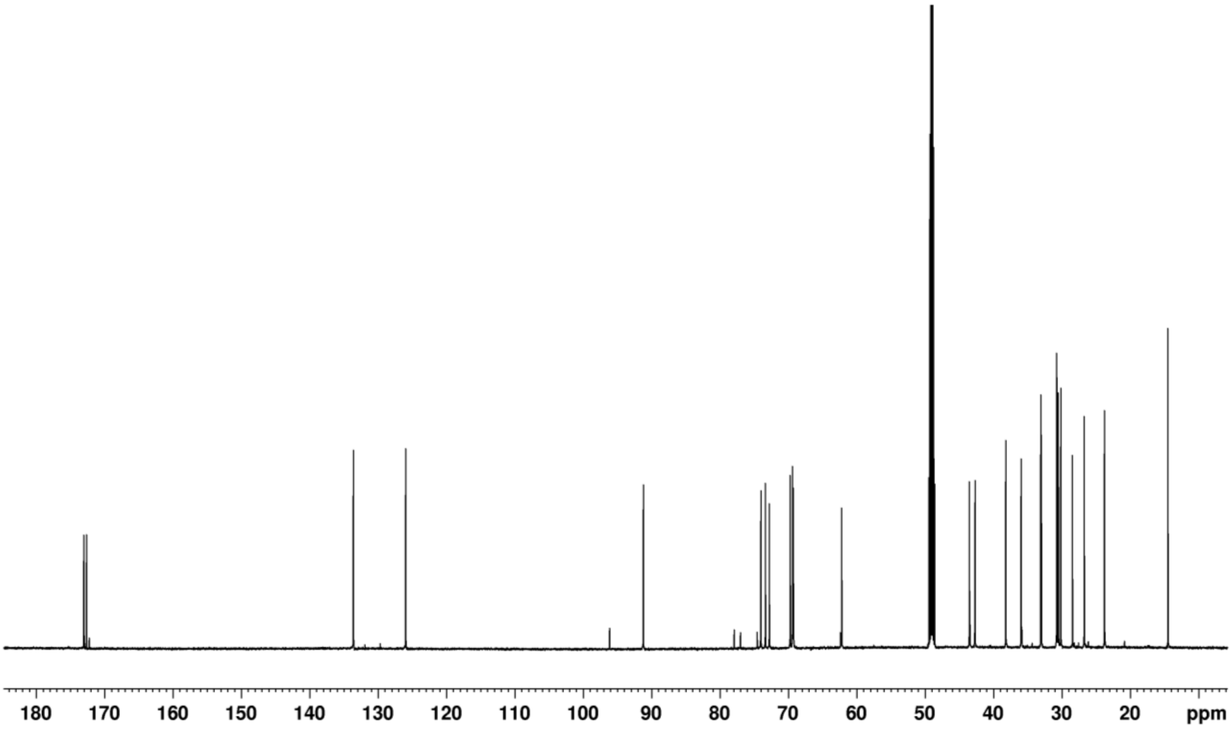

Figure S3 ^{13}C NMR spectrum of the glucose–lipid in methanol-d_4 at 150 MHz.

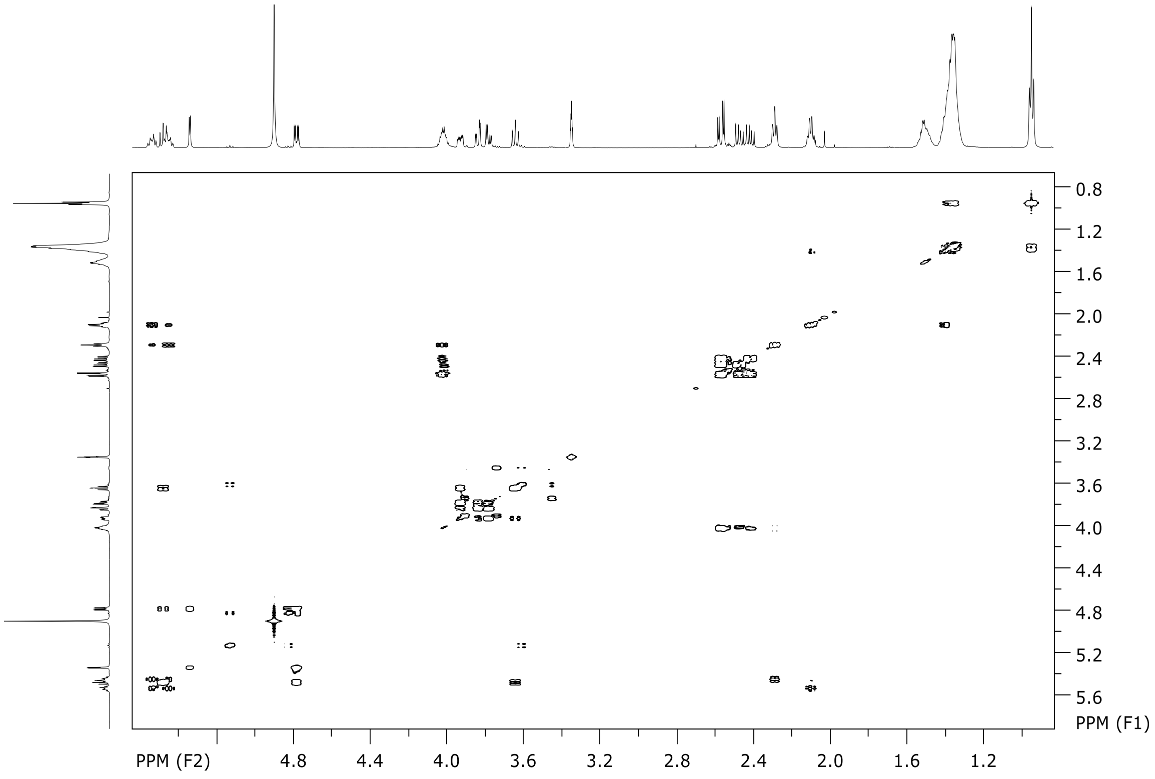

Figure S4 Gradient COSY spectrum of the glucose–lipid in methanol-d_4 at 600 MHz.

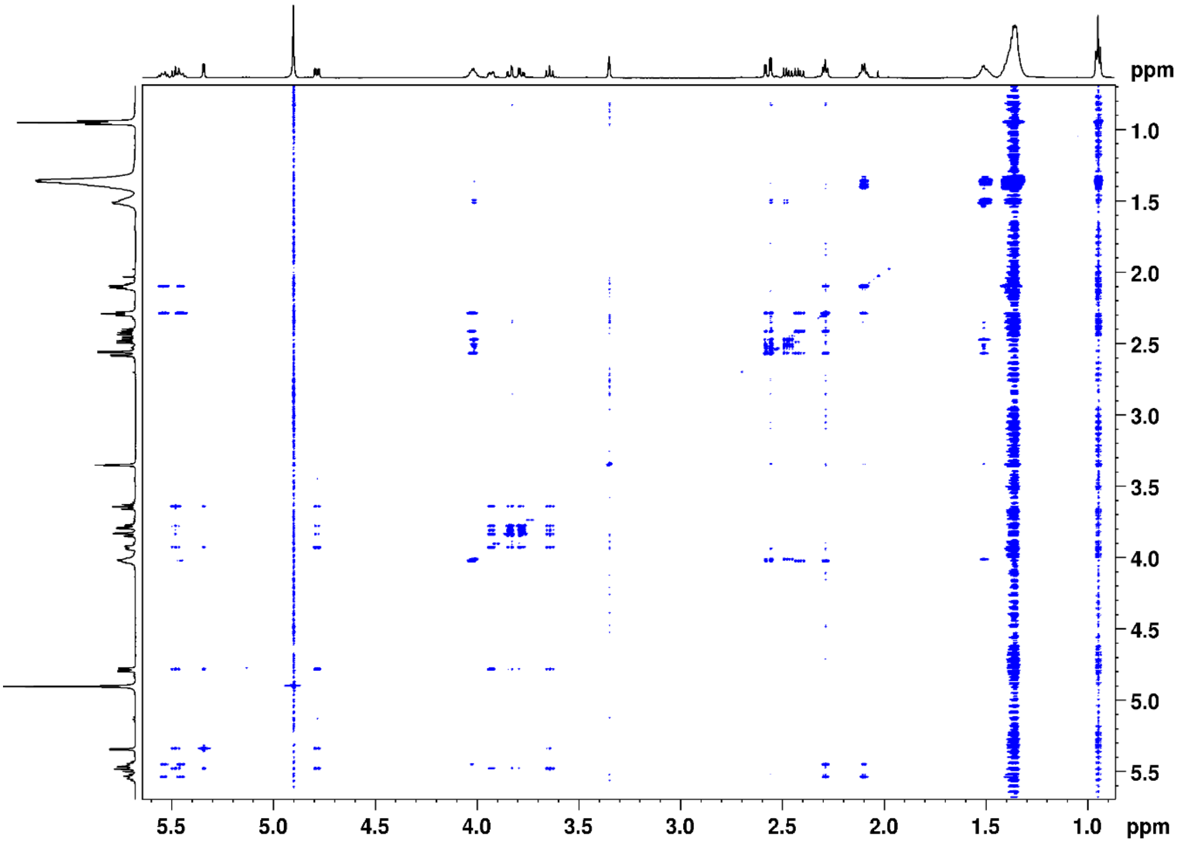

Figure S5 F1-homodecoupled PSYCHE TOCSY spectrum of the glucose–lipid in methanol-d_4 at 600 MHz.

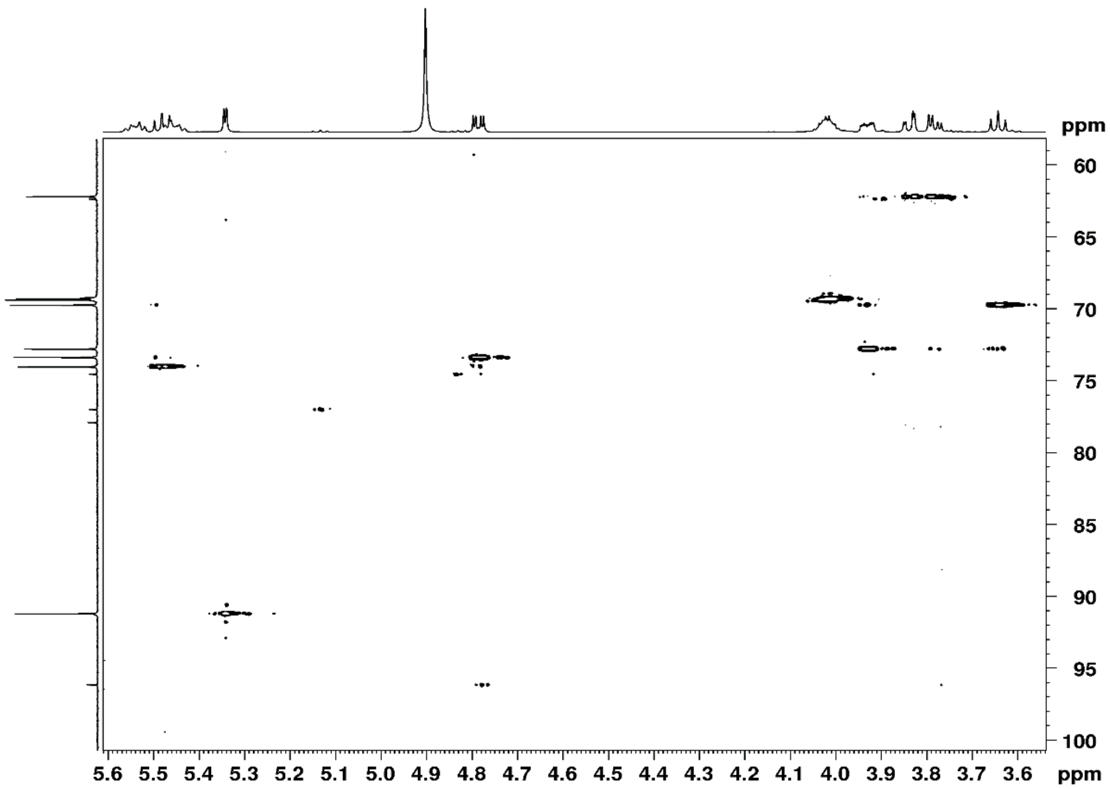

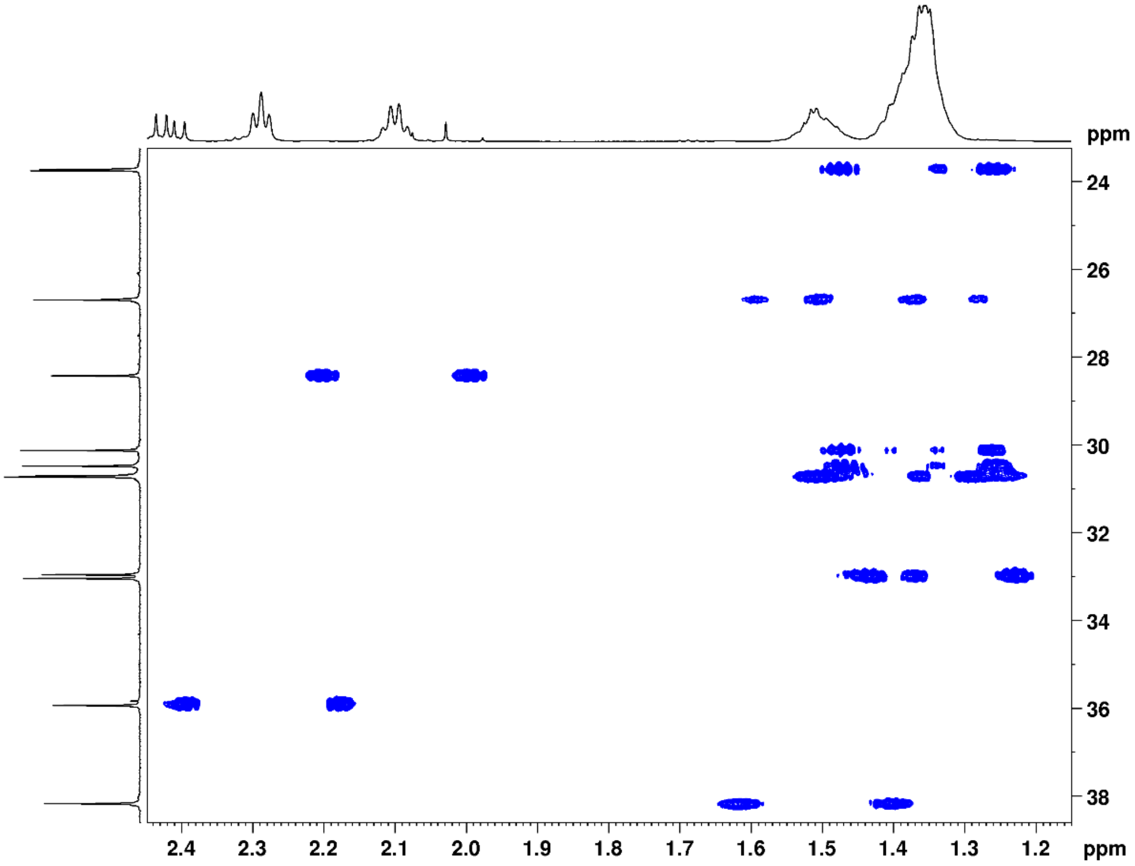

Figure S6 Expansion of the F1 homoband-decoupled HSQC spectrum of the glucose–lipid in methanol-d_4 at 600 MHz displaying the region of carbohydrate resonances (upper) and band-selective HSQC without decoupling to determine the J_{HH}-coupling constants of strongly overlapped signals (lower).

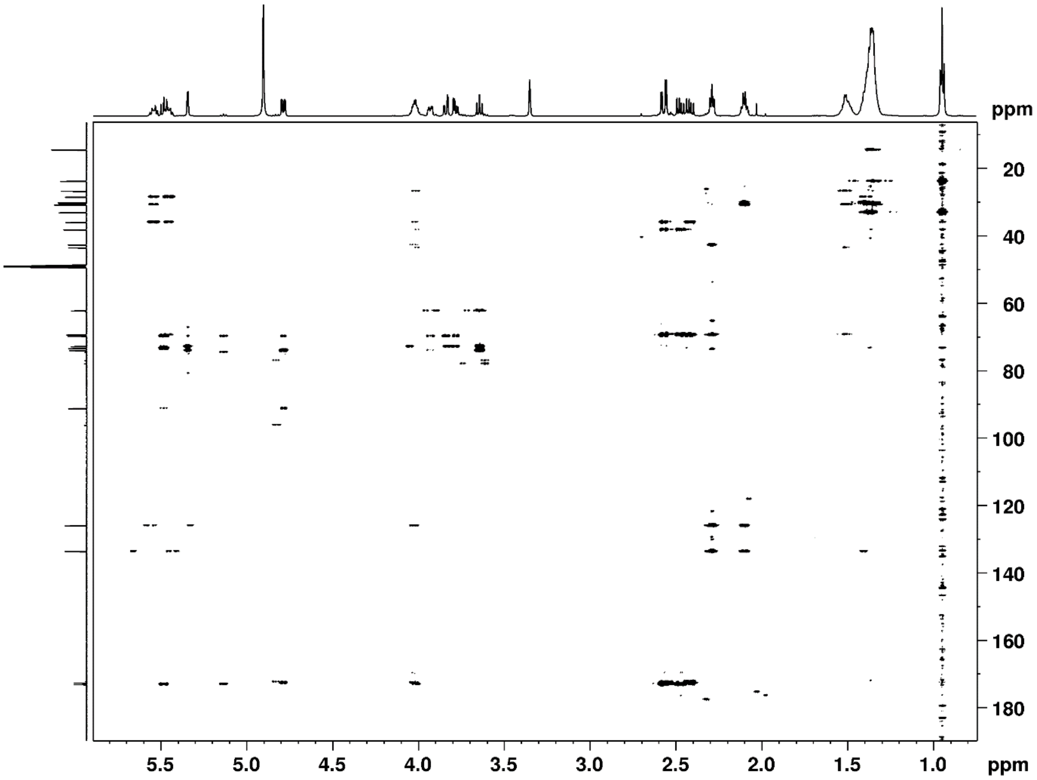

Figure S7 HMBC spectrum of the glucose–lipid in methanol-d_4 at 600 MHz.

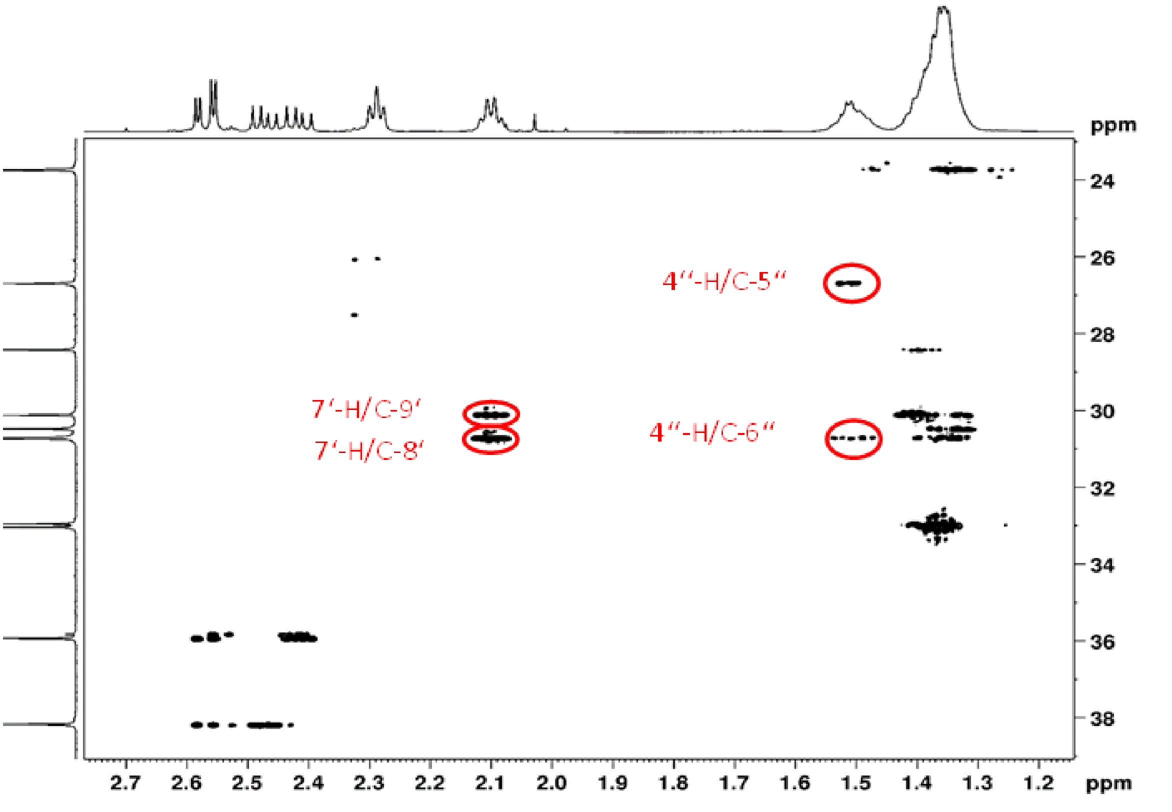

Figure S8 Band-selective HMBC spectrum of the glucose–lipid in methanol-d_4 at 600 MHz displaying the aliphatic region.

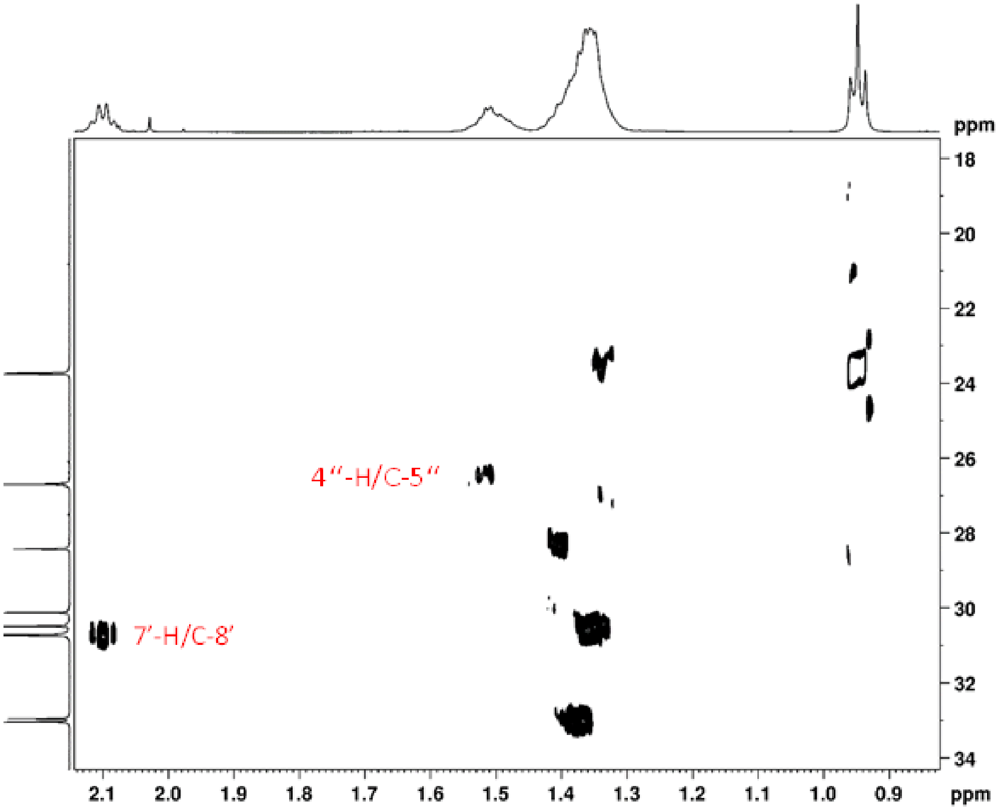

Figure S9 H2BC spectrum of the glucose–lipid in methanol-d_4 at 600 MHz displaying the aliphatic region.

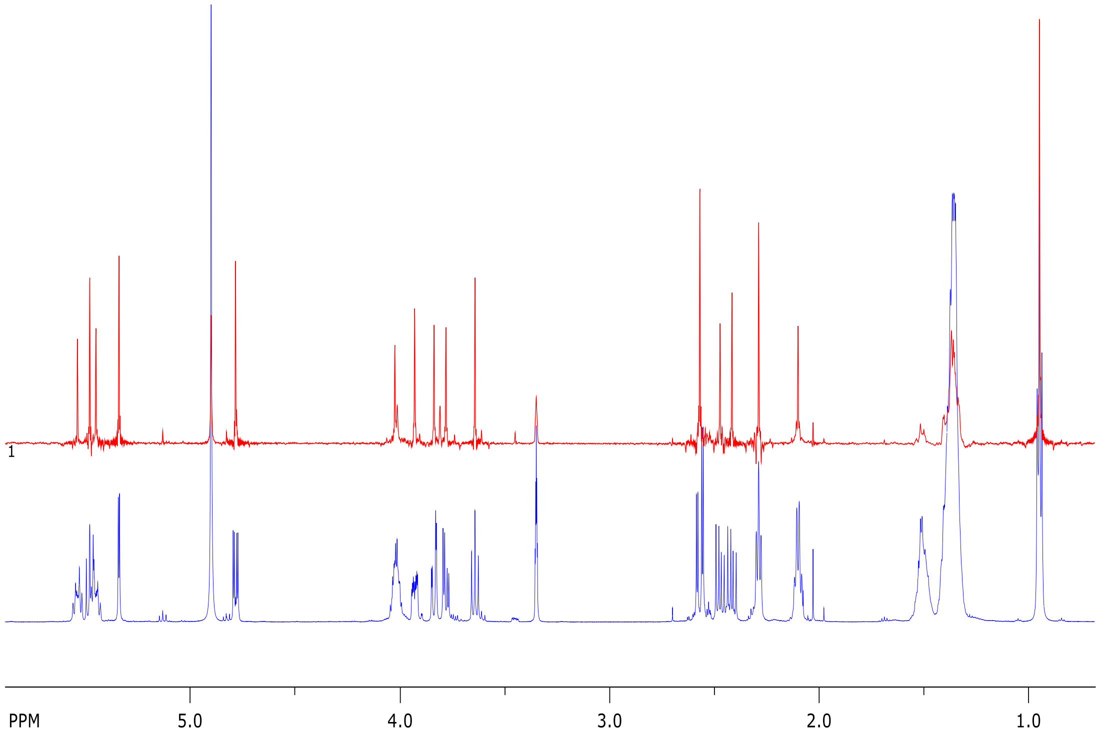

Figure S10 PSYCHE (upper trace) and ^{1}H NMR spectrum (lower trace) of the glucose–lipid in methanol-d_4 at 600 MHz.

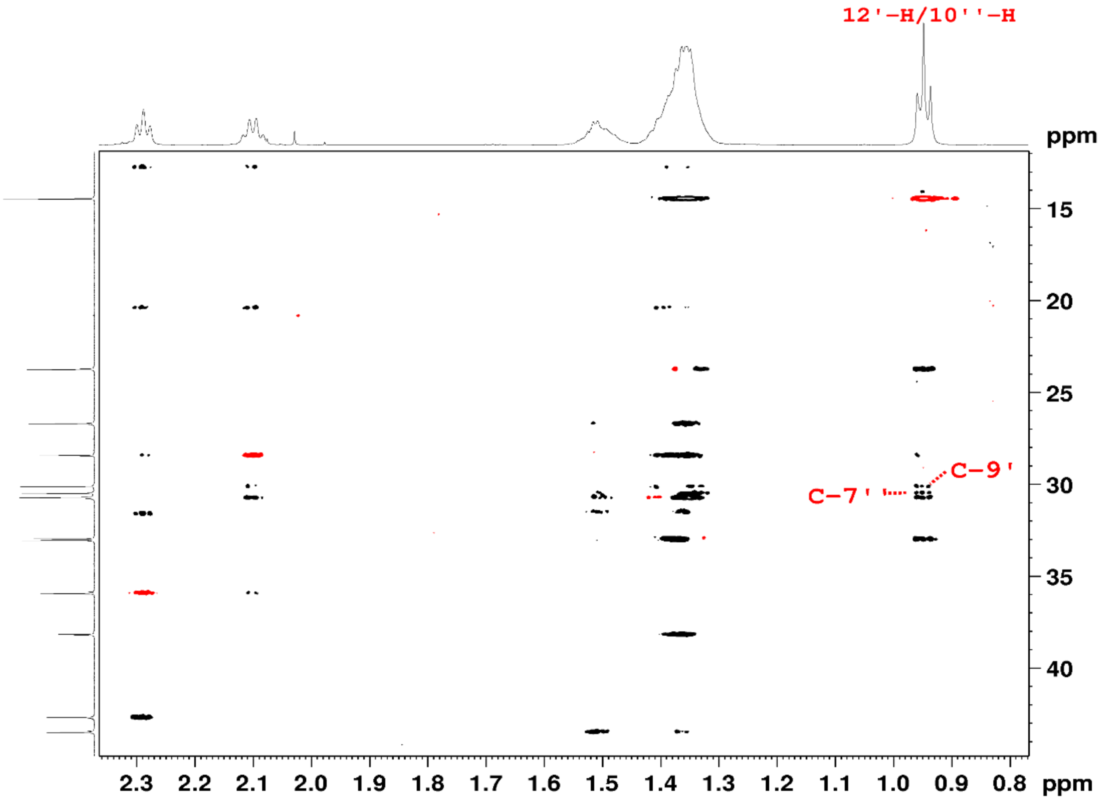

Figure S11 Expansion of the HSQCTOCSY spectrum of the glucose–lipid in methanol-d_4 at 600 MHz displaying the aliphatic region.

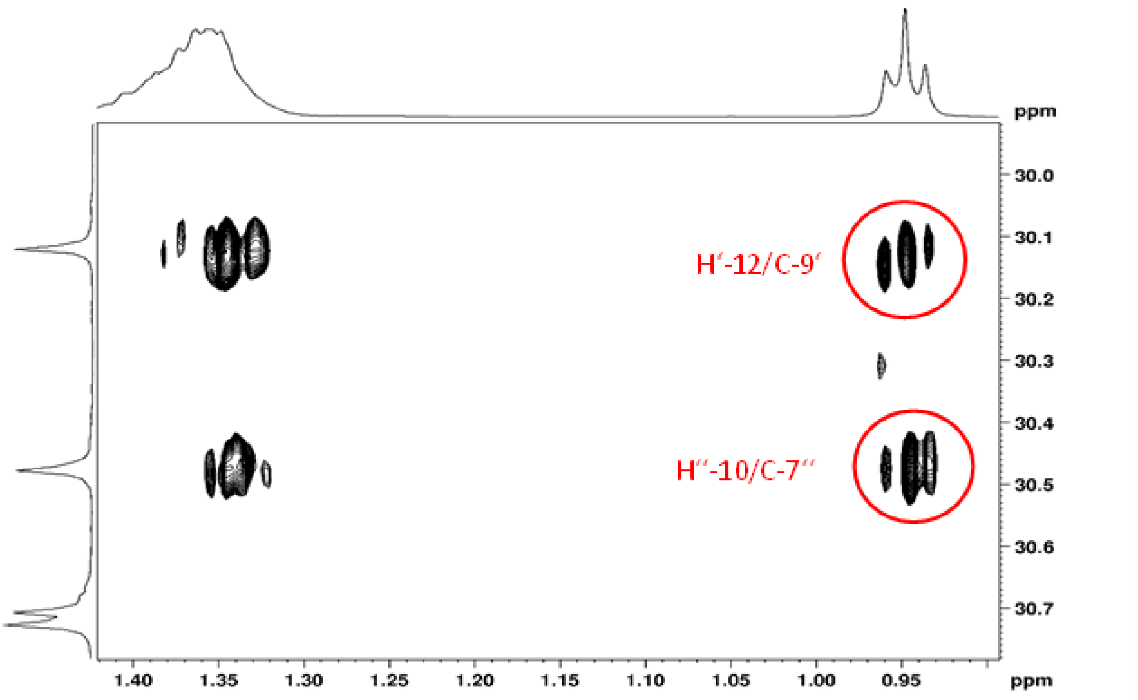

Figure S12 Expansion of the band-selective super-long-range HMBC spectrum of the glucose–lipid in methanol-d_4 at 600 MHz displaying $^4J_{CH}$ of terminal methyl groups of FAs.

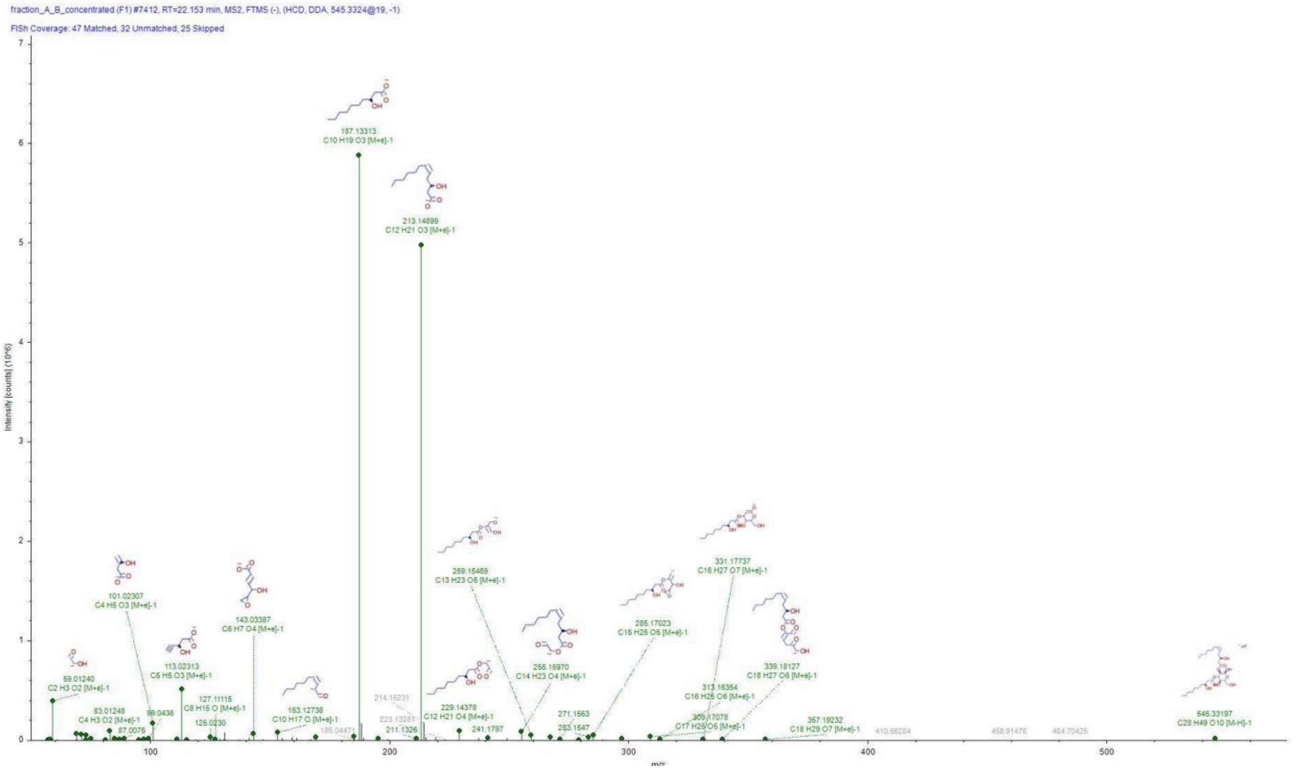

Figure S13 Fragment ion search (FISh) scoring in the negative ion mode MS/MS spectrum of m/z 545.3325 of the purified glucose–lipid sample. The theoretical fragmentation of the glycolipid structure determined by NMR analysis is in excellent agreement with the acquired MS/MS spectrum of m/z 545.3325 in negative ion mode.

Table S1. Determination of the conversion factor between cell dry mass and optical density in bioreactor cultivation (18 h) of *R. badensis* DSM 100043[T].

Sample	OD_{600} (-)	CDW (g/45 mL)	CDW (g/L)	OD_{600}/CDW	*c*	SD
A	11	0.17	3.85	2.86		
B	11	0.16	3.60	3.06	2.82	0.21
C	11	0.20	4.34	2.54		

OD_{600} : optical density at 600 nm
CDW : cell dry weight
c : conversion factor
SD : standard deviation

Table S2. Determination of the conversion factor between cell dry mass and optical density in shake flask cultivation of *R. badensis* DSM 100043[T].

Sample	Cultivation Time (h)	OD_{600} (-)	CDW (g/45 mL)	CDW (g/L)	OD_{600}/CDW	*c*	SD
Biological replicate A	4	0.9	0.03	0.62	1.45	2.81	0.25
	4	0.9	0.01	0.20	4.50		
	8	10.0	0.14	3.13	3.19		
	8	10.0	0.19	4.25	2.36		
	12	12.3	0.21	4.69	2.62		
	12	12.3	0.19	4.17	2.94		
Biological replicate B	4	1.0	0.02	0.44	2.25		
	4	1.0	0.02	0.49	2.04		
	8	10.2	0.17	3.76	2.72		
	8	10.2	0.17	3.71	2.75		
	12	12.3	0.18	3.97	3.09		
	12	12.3	0.20	4.42	2.78		

OD_{600} : optical density at 600 nm
CDW : cell dry weight
c : conversion factor
SD : standard deviation

Table S3. Overview of growth parameters showing $Y_{X/S}$, $Y_{P/S}$, and $Y_{P/X}$ for shake flask and bioreactor cultivations of *R. badensis* DSM 100043[T].

Parameter	Shake Flasks (15 g/L Glycerol)			Shake Flasks (30°C)			Bioreactor
	20 °C	25 °C	30 °C	10 g/L	20 g/L	30 g/L	
$Y_{X/S}$ (g/g)	0.38	0.40	0.32	0.44	0.27	0.28	0.37
$Y_{P/S}$ (g/g)	0.00428	0.00366	0.00815	0.00283	0.00247	0.00227	0.00273
$Y_{P/X}$ (g/g)	0.01373	0.01066	0.02839	0.00738	0.01014	0.00928	0.00866

Supplementary Information SM2

Structure Elucidation and Characterization of Novel Glycolipid Biosurfactant Produced by *Rouxiella badensis* DSM 100043^T

**Andre Fahriz Perdana Harahap [1], Jürgen Conrad [2], Mario Wolf [2], Jens Pfannstiel [3], Iris Klaiber [3], Jakob Grether [1], Eric Hiller [1], Maliheh Vahidinasab [1], Hanna Salminen [4], Chantal Treinen [5], Elvio Henrique Benatto Perino [1]
and Rudolf Hausmann [1,*]**

[1] Department of Bioprocess Engineering (150k), Institute of Food Science and Biotechnology,
University of Hohenheim, Fruwirthstr. 12, 70599 Stuttgart, Germany; andrefahrizperdana.harahap@uni-hohenheim.de (A.F.P.H.); jakob.grether@uni-hohenheim.de (J.G.); eric.hiller@uni-hohenheim.de (E.H.); malihe.vahidinasab@uni-hohenheim.de (M.V.);
eperino@uni-hohenheim.de (E.H.B.P.)

[2] Department of Organic Chemistry (130b), Institute of Chemistry, University of Hohenheim,
Garbenstr. 30, 70599 Stuttgart, Germany; juergen.conrad@uni-hohenheim.de (J.C.); wolf.mario@uni-hohenheim.de (M.W.)

[3] Mass Spectrometry Unit, Core Facility Hohenheim, University of Hohenheim, Ottilie-Zeller-Weg 2,
70599 Stuttgart, Germany; jens.pfannstiel@uni-hohenheim.de (J.P.); iris.klaiber@uni-hohenheim.de (I.K.)

[4] Department of Food Material Science (150g), Institute of Food Science and Biotechnology,
University of Hohenheim, Garbenstr. 21/25, 70599 Stuttgart, Germany; hanna.salminen@uni-hohenheim.de

[5] Cellular Agriculture, TUM School of Life Sciences, Technical University of Munich, 85354 Freising, Germany; chantal.treinen@tum.de

* Correspondence: rudolf.hausmann@uni-hohenheim.de

EXPERIMENT 1

Conc (mg/mL)	Volume (µL)	Volume (mL)	Mass (mg)	Mass (ng)	Area
0.1	5	0.005	0.0005	500	650.89
0.1	10	0.01	0.001	1000	953.93
0.1	15	0.015	0.0015	1500	1382.67
0.1	20	0.02	0.002	2000	1706.62
0.1	25	0.025	0.0025	2500	1945.64
0.1	30	0.03	0.003	3000	2365.3
0.1	35	0.035	0.0035	3500	2667.4
0.1	40	0.04	0.004	4000	2960.03

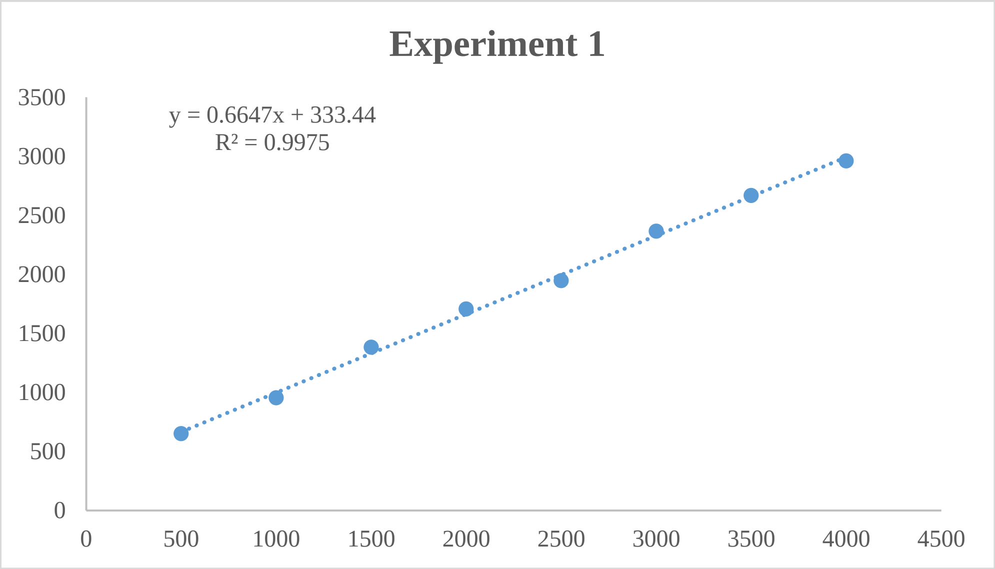

EXPERIMENT 2

Conc (mg/mL)	Volume (µL)	Volume (mL)	Mass (mg)	Mass (ng)	Area
0.1	5	0.005	0.0005	500	753.09
0.1	10	0.01	0.001	1000	1178.29
0.1	15	0.015	0.0015	1500	1437.51
0.1	20	0.02	0.002	2000	1759.77
0.1	25	0.025	0.0025	2500	2080.85
0.1	30	0.03	0.003	3000	2304.25
0.1	35	0.035	0.0035	3500	2606.26
0.1	40	0.04	0.004	4000	2875.25

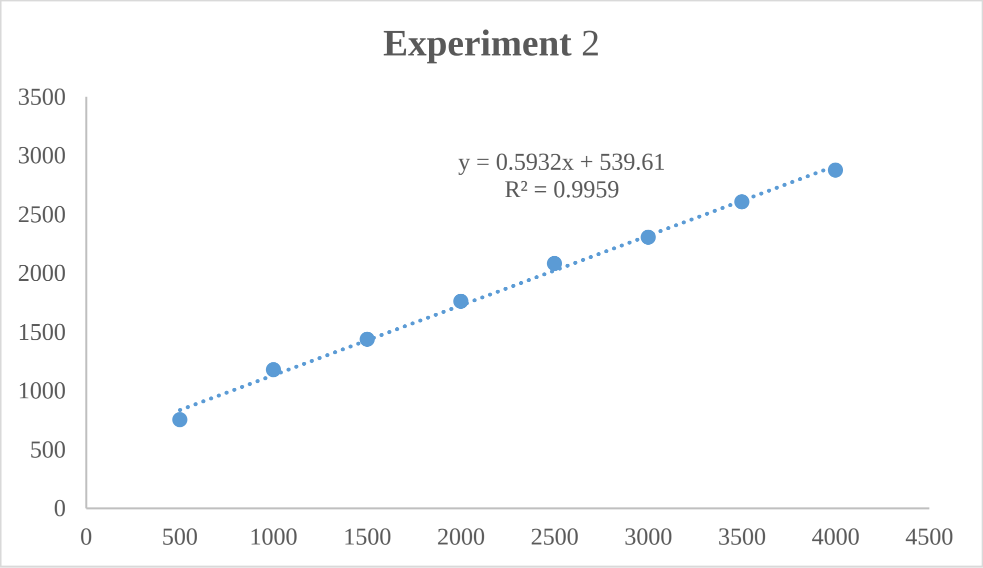

EXPERIMENT 3

Conc (mg/mL)	Volume (µL)	Volume (mL)	Mass (mg)	Mass (ng)	Area
0.1	5	0.005	0.0005	500	597.87
0.1	10	0.01	0.001	1000	874.12
0.1	15	0.015	0.0015	1500	1210.15
0.1	20	0.02	0.002	2000	1576.24
0.1	25	0.025	0.0025	2500	1854.71
0.1	30	0.03	0.003	3000	2146.63
0.1	35	0.035	0.0035	3500	2476.54
0.1	40	0.04	0.004	4000	2640.85

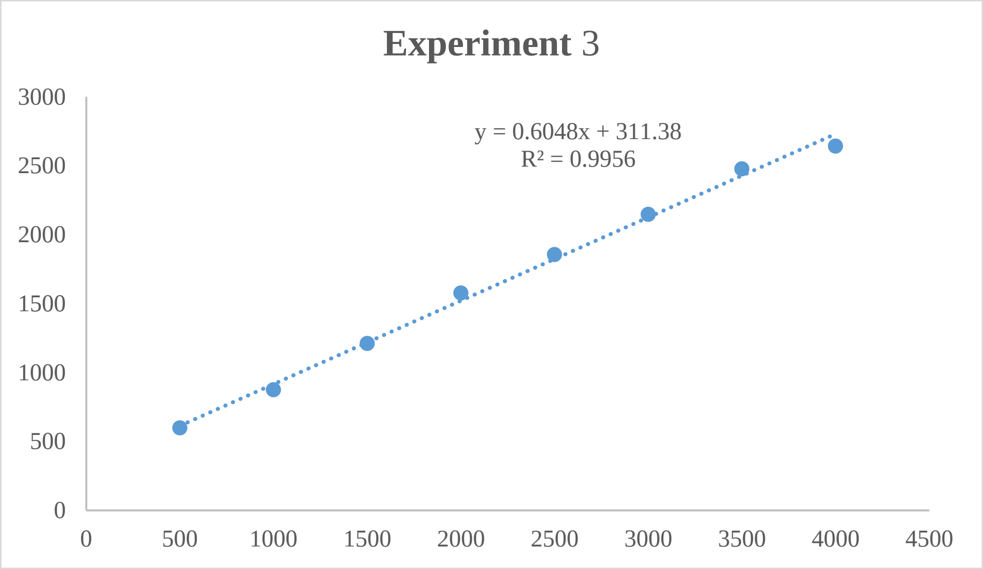

AVERAGE

Conc (mg/mL)	Volume (µL)	Volume (mL)	Mass (mg)	Mass (ng)	Area
0.1	5	0.005	0.0005	500	667.2833333
0.1	10	0.01	0.001	1000	1002.113333
0.1	15	0.015	0.0015	1500	1343.443333
0.1	20	0.02	0.002	2000	1680.876667
0.1	25	0.025	0.0025	2500	1960.4
0.1	30	0.03	0.003	3000	2272.06
0.1	35	0.035	0.0035	3500	2583.4
0.1	40	0.04	0.004	4000	2825.376667

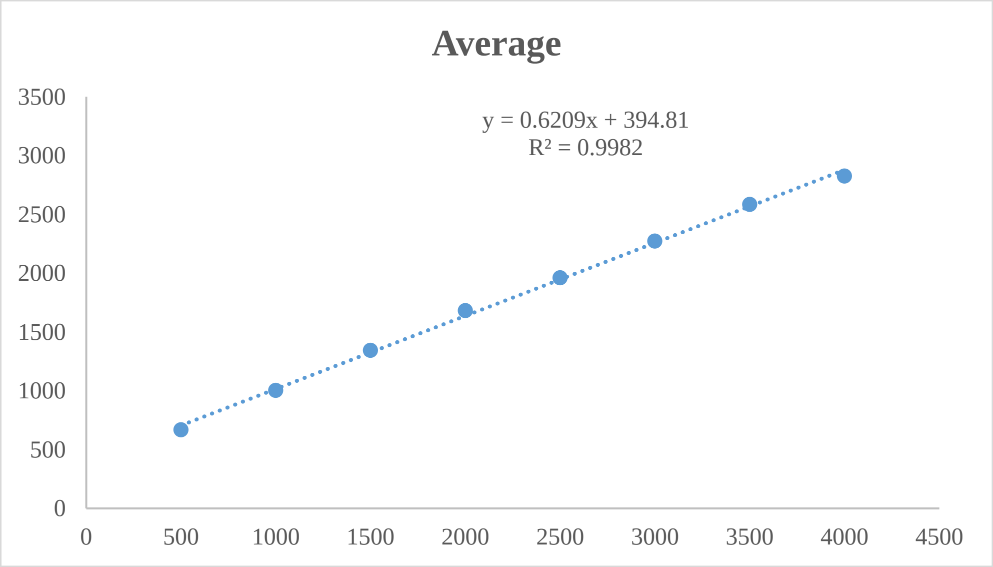

**Standard error**	27.22707387
**Standard deviation**	77.00979427
**LOD (ng/zone)**	409
**LOQ (ng/zone)**	1240

ISSUE: SUMMARY

Regression Statistics	
Multiple correlation coefficient	0.999096374
Coefficient of determination	0.998193564
Adjusted coefficient of determination	0.997892492
Standard error	34.94262478
Observations	8

ANOVA

	Degrees of Freedom (df)	*Sums of Squares (SS)*	*Mean Sum of Squares (MS)*	*Test Size (F)*	*F test*
Regression	1	4048131.405	4048131.405	3315.458164	1.84337E-09
Residue	6	7325.922157	1220.987026		
Total	7	4055457.327			

	Coefficient	*Standard Error*	*t-Statistic*	*P-Value*	*Under 95%*	*Over 95%*	*Under 95.0%*	*Over 95.0%*
Intersection	394.8088095	27.22707387	14.50059641	6.74376E-06	328.1865598	461.4310593	328.1865598	461.431059
X variable 1	0.620915714	0.010783528	57.58001532	1.84337E-09	0.594529372	0.647302057	0.594529372	0.64730206

Samples	Concentration	Unit	Concentration	Unit
A32	64.31	mg/L	0.06431	mg/mL
C32	83.71	mg/L	0.08371	mg/mL
F32	49.79	mg/L	0.04979	mg/mL
B32	55.97	mg/L	0.05597	mg/mL
Standard	0.1	mg/mL		
**Vol. sample**	**0.1**	**mL**		

Spiked Samples	Concentration	Unit	Concentration	Unit
A32 + 0.1 mL standard	0.082155	mg/mL	82.16	mg/L
C32 + 0.15 ml standard	0.093484	mg/mL	93.48	mg/L
C28 + 0.2 mL standard	0.083263	mg/mL	83.26	mg/L
B32 + 0.25 mL standard	0.087420	mg/mL	87.42	mg/L

PRECISSION CHECK

Spiked Sample	Concentration (mg/L)				SD	REL. SD (%)
	n1	n2	n3	Mean		
A32'	74.28	84.87	79.93	79.69	5.30	6.65
C32'	88.36	92.17	98.20	92.91	4.96	5.34
C28'	78.77	92.62	89.28	86.89	7.23	8.32
B32'	80.49	82.22	92.53	85.08	6.51	7.65

REL. SD < 15%

ACCURACY CHECK

Spiked Samples	Expected Conc. (mg/L)	Measured Conc. (mg/L)	Recovery (%)
A32'	82.16	79.69	97.00
C32'	93.48	92.91	99.39
C28'	83.26	86.89	104.36
B32'	87.42	85.08	97.32

Difference < ±15%

REPEATABILITY CHECK

Conc (mg/mL)	Volume (µL)	Volume (mL)	Mass (mg)	Mass (ng)	
0.1	10	0.01	0.001	1000	Standard A
0.1	40	0.04	0.004	4000	Standard B

		INTRADAY															INTERDAY		
		DAY 1					DAY 2					DAY 3							
		n1	n2	Mean	SD	RSD (%)	n1	n2	Mean	SD	RSD (%)	n1	n2	Mean	SD	RSD (%)	Mean	SD	RSD (%)
Standard A	1000 ng/zone	1129	993	1061	96	9.06	997	958	977.5	28	2.82	915	868	891.5	33	3.73	977	85	8.68
Standard B	4000 ng/zone	2932	2827	2879.5	74	2.58	2883	2746	2814.5	97	3.44	2810	2703	2756.5	76	2.74	2817	62	2.18
